# Discovery and validation of candidate smoltification gene expression biomarkers across multiple species and ecotypes of Pacific salmonids

**DOI:** 10.1093/conphys/coz051

**Published:** 2019-10-11

**Authors:** Aimee Lee S Houde, Oliver P Günther, Jeffrey Strohm, Tobi J Ming, Shaorong Li, Karia H Kaukinen, David A Patterson, Anthony P Farrell, Scott G Hinch, Kristina M Miller

**Affiliations:** 1 Department of Forest and Conservation Sciences, University of British Columbia, Vancouver, British Columbia, V6T 1Z4, Canada; 2 Pacific Biological Station, Fisheries and Oceans Canada, Nanaimo, British Columbia, V9T 6N7, Canada; 3 Günther Analytics, 402-5775 Hampton Place, Vancouver, British Columbia, V6T 2G6, Canada; 4 School of Resource and Environmental Management, Fisheries and Oceans Canada, Simon Fraser University, Burnaby, British Columbia, V5A 1S6, Canada; 5 Department of Zoology and Faculty of Land and Food Systems, University of British Columbia, Vancouver, British Columbia, V6T 1Z4, Canada

**Keywords:** Aquaculture, de-smolt, hatchery, Na^+^/K^+^-ATPase activity, parr–smolt transformation, transcription

## Abstract

Early marine survival of juvenile salmon is intimately associated with their physiological condition during smoltification and ocean entry. Smoltification (parr–smolt transformation) is a developmental process that allows salmon to acquire seawater tolerance in preparation for marine living. Traditionally, this developmental process has been monitored using gill Na^+^/K^+^-ATPase (NKA) activity or plasma hormones, but gill gene expression offers the possibility of another method. Here, we describe the discovery of candidate genes from gill tissue for staging smoltification using comparisons of microarray studies with particular focus on the commonalities between anadromous Rainbow trout and Sockeye salmon datasets, as well as a literature comparison encompassing more species. A subset of 37 candidate genes mainly from the microarray analyses was used for TaqMan quantitative PCR assay design and their expression patterns were validated using gill samples from four groups, representing three species and two ecotypes: Coho salmon, Sockeye salmon, stream-type Chinook salmon and ocean-type Chinook salmon. The best smoltification biomarkers, as measured by consistent changes across these four groups, were genes involved in ion regulation, oxygen transport and immunity. Smoltification gene expression patterns (using the top 10 biomarkers) were confirmed by significant correlations with NKA activity and were associated with changes in body brightness, caudal fin darkness and caudal peduncle length. We incorporate gene expression patterns of pre-smolt, smolt and de-smolt trials from acute seawater transfers from a companion study to develop a preliminary seawater tolerance classification model for ocean-type Chinook salmon. This work demonstrates the potential of gene expression biomarkers to stage smoltification and classify juveniles as pre-smolt, smolt or de-smolt.

## Introduction

Beyond their cultural importance, salmonids can provide over a billion dollars annually to the economies of countries with recreational and commercial fisheries (e.g. Canada; Pinfold, 2011). Yet, populations of several salmonid species are declining on the Pacific and Atlantic coasts, and a lower than historical survival of juveniles during their early marine phase is associated with these declines (Friedland *et al*., 2003; Beamish *et al*., 2010; Mills *et al*., 2013). To increase salmonid populations and augment fisheries, hatchery breeding programs are used (Fraser, 2008). As well, aquaculture is used to alleviate some of the fishing pressure on wild populations (Naylor *et al*., 2000) and provide additional economic opportunities (Bostock *et al*., 2010). However, the success of both hatcheries and aquaculture is known to be limited by the physiological condition of the smolt life stage during the transition from freshwater to seawater (e.g. Chittenden *et al*., 2008; Stien *et al*., 2013). Consequently, tools to measure the physiological condition of smolts are routinely used and improvements in them sought to inform culture and decisions for optimizing smolt performance.

All salmonid species begin their lives in freshwater as eggs, alevins, fry and parr, then the anadromous forms become smolts for a successful outmigration to seawater, where rapid somatic growth and increased reproductive success are greatly improved over freshwater residence. A trade-off may be lower survival because of increased predation, variable prey availability and other risks in the marine environment (Quinn, 2005). The developmental process preparing salmonids for the transition from freshwater to marine habitats is termed smoltification or parr–smolt transformation, which is characterized by changes in behaviour, skin pigmentation, body morphology and physiology (reviewed by McCormick *et al*., 1998, 2013; Björnsson *et al*., 2011). Changes in behaviour include increased negative rheotaxis (i.e. downstream movement) and schooling (i.e. the loss of territorial behaviour). The schooling behaviour may lower the risks of predation in river and the early marine environment. Changes in skin pigmentation include acquiring silver skin pigmentation and dark caudal fin tips. Changes in body morphology include a more streamlined body shape, elongation of the caudal peduncle and associated lower body condition and increased buoyancy. These changes in pigmentation and morphology may be adaptations to marine habitats, providing camouflage from predators and increasing swimming performance in open water, respectively.

The physiological changes during smoltification are equally numerous, such as red blood cell hemoglobin isoforms, increased metabolic rate and seawater tolerance. Changes in hemoglobin isoforms from juvenile to adult types may increase the oxygen affinity of the blood (Vanstone *et al*., 1964). Higher metabolic rate may be to meet the increased energetic demands during smoltification and migration (Robertson and McCormick, 2012). Of the physiological changes, the acquired seawater tolerance may be the most important for immediate survival (McCormick *et al*., 1998, 2013; Björnsson *et al*., 2011). Indeed, juvenile salmonids that are unprepared for increased salinity, i.e. pre-smolts that have not completed the parr–smolt transformation or de-smolts that have remained in freshwater too long and have reverted to a physiology more suited to freshwater, have greatly reduced survival and slower growth because of internal ionic and osmotic disturbances from the excess ions in seawater relative to freshwater. Nevertheless, seawater tolerance is limited in its duration and is often referred to as a ‘smoltification window’, one that may narrow with elevated water temperature, which may have implications with global climate change (e.g. Bassett *et al*., 2018). Moreover, hatchery-reared juveniles generally have a lower seawater tolerance than wild juveniles (e.g. Shrimpton *et al*., 1994; Chittenden *et al*., 2008), suggesting that the smoltification window may be altered in a culture environment. Consequently, knowing the smolt status of juveniles in particular is crucial to optimize the timing of smolt release for hatchery and aquaculture operations. Altogether, hatcheries and aquaculture can benefit from tools that reliably measure the smolt status of salmonids for planning releases and modifying the culture environment, if necessary.

In general, existing tools take advantage of known changes associated with smotification. For example, salmonids generally need to reach a critical body size prior to smoltification. Photoperiod and, to a lesser extent, temperature also drives smoltification (McCormick *et al*., 2002). Since an increase in day length activates the light–brain–pituitary axis to release a cascade of hormones including growth hormone, insulin-like growth factor I, cortisol and thyroid hormone, these hormones can be monitored in plasma samples. Growth hormone and cortisol stimulate the development of gill ionocytes and their associated Na^+^/K^+^-ATPase (NKA; McCormick, 1993; Evans *et al*., 2005), the activity of which can be monitored in gill samples. Thyroid hormones may be involved in the changes in behaviour and skin pigmentation, which are useful visual indicators of smoltification. All the same, smoltification research has mainly focused on species and ecotypes that migrate to seawater after one or more years in freshwater, e.g. Coho salmon (*Oncorhynchus kisutch*), stream-type Chinook salmon (*Oncorhynchus tshawytscha*; see Bourret *et al*., 2016), Sockeye salmon (*Oncorhynchus nerka*), anadromous Rainbow trout (*Oncorhynchus mykiss*), Atlantic salmon (*Salmo salar*) and Brook trout (*Salvelinus fontinalis*). However, species and ecotypes that migrate to the ocean after less than a year in freshwater, e.g. ocean-type Chinook salmon (*O. tshawytscha*, see Bourret *et al*., 2016), Pink salmon (*Oncorhynchus gorbuscha*), and Chum salmon (*O. keta*), enter seawater at a smaller body size and may remain longer in estuaries than the other groups. In these species and ecotypes, smoltification may not depend on photoperiod and may be body size based (Clarke *et al*., 1992, 1994; Gallagher *et al*., 2013, but see Hoffnagle and Fivizzani, 1998). Thus, tools to define smolt status have focused on gill NKA activity and plasma hormone concentrations.

Recently, techniques for monitoring smoltification have shifted to candidate gill gene expression using quantitative PCR (qPCR) for hormones and their receptors (e.g. Kiilerich *et al*., 2007; Hecht *et al*., 2014), as well as the precursors to NKA (e.g. Nilsen *et al*., 2007; Piironen *et al*., 2013). In particular, the gill expression of NKA α-1 isoforms for ‘a’ freshwater and ‘b’ seawater ion regulation (c.f. Richards *et al*., 2003; Shrimpton *et al*., 2005), which typically change reciprocally during smoltification, are compared. More recently, smoltification has been examined at the genomic level using microarrays (e.g. Seear *et al*., 2010; Robertson and McCormick, 2012; Sutherland *et al*., 2014), which have identified gill expression patterns for the upregulation of biological functions such as ion regulation, metabolic rate, oxygen transport, growth, structural integrity (e.g. collagen), calcium uptake (i.e. nutrient limitation for growth) and immunity, as well as downregulation of immunity and a few ion regulation and hormones. The upregulation of innate immunity is suggested as a preparation for exposure to new pathogens in marine environments (Boulet *et al*., 2012), while the downregulation of anti-viral immunity (Sutherland *et al*., 2014) is suggested to reflect suppression by elevated cortisol (Lemmetyinen *et al*., 2013). Despite these recent advances, it is not known if expression patterns of specific genes for smoltification can be reliably applied across salmonid species and different ecotypes.

Therefore, our objective was to discover candidate genes for smoltification and validate a subset of these genes using new samples from multiple species with different ecologies. To this end, we used mapping approaches to discover candidate smoltification genes by a meta-analysis of microarray gene expression patterns across studies. In particular, we focused on a comparison between anadromous Rainbow trout (Sutherland *et al*., 2014) and in-house Sockeye salmon datasets, as well as mining the literature for a wider collection of salmonid studies based on gene names. We then selected a subset of candidate genes for validation. These genes were developed into TaqMan qPCR assays and tested for expected gene expression patterns using gill samples from Coho salmon, Sockeye salmon, stream-type Chinook salmon and ocean-type Chinook salmon of various hatchery and wild sources. We used the Fluidigm BioMark™ HD platform for measuring gene expression, a high-throughput microfluidics-based technology that can individually quantify 96 assays across 96 samples at once. We focused on these four groups because of their population declines in Southern British Columbia (BC), Canada and subsequent hatchery supplementation (Noakes *et al*., 2000; Beamish *et al*., 2009; DFO, 2013). In particular, the Sockeye salmon were from the endangered population of Cultus Lake, BC (COSEWIC, 2003). Beyond the gill smoltification biomarkers, we are also developing biomarkers predictive of other divergent stressors, e.g. general stress and imminent mortality (Evans *et al*., 2011; Miller *et al*., 2011; Jeffries *et al*., 2012, 2014); viral disease development (Miller *et al*., 2017); and salinity, thermal and hypoxic stress (Houde *et al*., 2019), to support the development of a ‘Salmon Fit-Chip’ tool to rapidly and inexpensively assess the physiological condition of hundreds to thousands of fish at one time.

We hypothesize that a suitable panel of biomarkers will show a consistent association with the smoltification process across species and ecotypes, and the specific level of activation of this smoltification biomarker panel alone could predict smolt status. As such, the present study would mark the first step in a process by identifying biomarkers that change with smolt development. Our companion study examines the gene expression associated with seawater survival using pre-smolt, smolt and de-smolt juveniles (e.g. ocean-type Chinook salmon; Houde *et al*., 2019). Using the smolt status for the trials of the companion study, here we explore a preliminary seawater tolerance classification model for ocean-type Chinook salmon. We examined how the seawater tolerance changed during development for ocean-type Chinook salmon in the present study.

## Materials and methods

### Candidate smoltification gene discovery

Smoltification candidate genes for gill tissue were identified using two approaches: (i) comparisons between a Sockeye salmon (*O. nerka*) cGRASP 44K internal microarray dataset of the Molecular Genetics Lab, Pacific Biological Station, Nanaimo, BC and the signatures of four external cGRASP microarray studies, i.e. 44K: Sutherland *et al.* (2014) and 16K: Robertson and McCormick (2012), Boulet *et al*. (2012), Lemmetyinen *et al*. (2013), and (ii) a literature mining of significant gene names across published studies. Statistical analyses were conducted in R 3.1.2 (R Core Team). Methods for the Sockeye salmon microarray studies are described by Miller *et al*. (2009, 2011). The Sockeye salmon dataset is composed of seven parr and eight smolt samples for 27 104 features. This dataset was filtered with a 50% threshold for missing values and imputation of missing values was performed with the mean value over available samples. The Rainbow trout dataset (Sutherland *et al*., 2014) was downloaded from National Center for Biotechnology Information (NCBI)’s Gene Expression Omnibus (GEO) public repository using the *GEOquery* R package (Sean and Meltzer, 2007) and the processing steps of the authors were honoured.

For the direct comparisons between the internal Sockeye salmon and external microarray datasets, first significant features that separated parr and smolt for the Sockeye salmon dataset were identified using the robust empirical Bayes method of the *limma* R package (Ritchie *et al*., 2015). Features with a false discovery rate < 0.05 were considered significant. Next, to identify the top 100 features that separated parr and smolt for both species, significant features of the Rainbow trout and the Sockeye salmon datasets (both 44K platforms) were combined and analysed collectively using a sparse independent principal component analysis with the *mixOmics* R package (Rohart *et al*., 2017). These 100 features were examined for overlap with the identified significant features from the Sockeye salmon robust limma analysis described above. For the remaining three datasets using the 16K platform, both the 16K and 44K features were mapped to Atlantic salmon gene IDs from NCBI (see Supplementary Methods), enabling comparisons across platforms. Similarly, the 16K features were examined for overlap with the identified significant features for both Sockeye salmon and Rainbow trout datasets.

Mining published literature involved discovering the overlap of significant gene names across five microarray studies that used the gill tissue of salmonid fishes, i.e. the four external microarray studies and Seear *et al*. (2010) that used a TRAITS/SGP microarray. The study tables were visually examined for overlap using generalized gene names given the relatively few studies and the multiple but different gene subunits contributing to a protein, so that names significantly separating parr and smolt in at least two microarray studies could be recorded and attributed a smoltification function. Five additional candidate gene studies (i.e. Kiilerich *et al*., 2007; Nilsen *et al*., 2007; Stefansson *et al*., 2007; Piironen *et al*., 2013; Hecht *et al*., 2014) examining the expression of specific ion regulation, hormone and hormone receptor genes for gill tissue were also considered.

### Validation samples

Juveniles from four groups (three species and two ecotypes) were collected monthly between November 2015 to May 2016, a time period spanning the smoltification period at four Salmon Enhancement Program hatchery facilities: Nitinat Hatchery and Quinsam Hatchery on Vancouver Island, BC for Coho salmon and ocean-type Chinook salmon (Table 1), Inch Creek Hatchery and Chehalis Hatchery on mainland BC for Sockeye salmon and stream-type Chinook salmon, respectively. In addition, wild (i.e. natural-born) juvenile counterparts of Coho salmon and Sockeye salmon were collected from the hatchery-supplemented source rivers and lakes using baited traps, dip nets, seines or downstream fences. We targeted 20–30 individuals monthly for each set and the last collection date was as close as possible to the hatchery release date. Federal hatcheries guidelines in BC suggest that the time of release should coincide with that of the wild migration (MacKinlay *et al*., 2004), but certain hatcheries may have a specific range of dates used every year—both strategies are presumed to be in line with smoltification.

**Table 1:** Summary of samples sizes for the four groups collected from four hatcheries and their wild source counterpart

	November	December	January	February	March	Early April	Late April	May
**Nitinat Hatchery**								
Coho salmon (Nitinat River), age 1+								
Hatchery	20	-	20	20	20	20	-	20
Wild	30	-	30	30	30	30	-	20
Chinook salmon (Nitinat River), age 0+								
Hatchery	-	-	20	20	20	20	20, 20 E	20 E
Chinook salmon (Sarita River), age 0+								
Hatchery	-	-	20	20	20	20	-	20
**Quinsam Hatchery**								
Coho salmon (Quinsam River), age 1+								
Hatchery	30	-	30	30	30	30		30
Wild	30	-	30	35	30	29		30
Chinook salmon (Quinsam River), age 0+								
Hatchery	-	-	-	30	30	30		30
**Inch Creek Hatchery**								
Sockeye salmon (Cultus Lake), age 1+								
Hatchery	20	20	20	20	20	20		
Wild	20	-	-	20	17	10		
**Chehalis Hatchery**								
Chinook salmon (Chilko River), age 1+								
Hatchery		20	20	19	20	30		
Chinook salmon (Upper Fraser Summer Red), age 1+								
Hatchery		20	20	20	20	20		

Presented is the number of juveniles of Coho salmon (*O. kisutch*), Sockeye salmon (*O. nerka*), Chinook salmon (stream-type, *O. tshawytscha*) and Chinook salmon (ocean-type, *O. tshawytscha*). Upper Fraser Summer Red is a mixture of Slim Creek and Chilliwack River origin fish. Juveniles were collected from freshwater unless denoted by the symbol ‘E’, which denotes juveniles from an estuary where they were exposed to seawater for about 2 weeks. Digital photographs were collected for the Nitinat and Quinsam juveniles in March, April and May. Nitinat wild Coho salmon were collected from Campass Creek, a neighbouring tributary of Nitinat River, which was smaller and thus more feasible for catching juveniles with traps than Nitinat River.

Fish were euthanized using buffered MS-222 (300 mg L^−1^) then measured for length (±0.1 cm) and mass (±0.01 g). Body condition was calculated as 100 × mass ÷ length^3^ (Fulton, 1904). For the months of March, April and May, Nitinat and Quinsam hatchery and wild juveniles were also digitally photographed (Nikon Coolpix AW110) using a camera stand with a light grey background and a length scale. Photographs were examined for skin pigmentation and body morphology (detailed by Houde *et al*., 2015) to generate LAB colour space values for anterior, posterior and caudal fin regions, which were subjected to a principal component analysis, as well as morphology values using 21 landmarks, which were subjected to a relative warp analysis using ‘tpsRelw32’ software (Rohlf, 2017). Gill tissue from the right side was then placed into a cryovial and immediately frozen with liquid nitrogen or dry ice for NKA activity. Gill tissue from the left side (used for gene expression) was placed into RNAlater (Ambion) for 24 h before freezing or the whole fish was placed into a Whirl-Pak bag and then immediately frozen between slabs of dry ice for later gill dissection. Tissues were stored at −80°C until used for measurements.

### Gene expression

A minimum subset of eight individual fish were targeted each month for gill gene expression and were measured for NKA activity (McCormick, 1993) in around half of these samples. For gene expression, gill tissue was homogenized in TRIzol (Ambion) and BCP reagent using stainless steel beads on a MM301 mixer mill (Retsch Inc.). RNA was extracted from the homogenate using the ‘No-Spin Procedure’ of MagMAX-96 Total RNA Isolation kits (Ambion) and a Biomek FXP automation workstation (Beckman-Coulter). RNA yield was quantified using the A_260_ value and extracts were normalized to 62.5 ng ml^−1^. Normalized RNA was reverse transcribed to cDNA using SuperScript VILO synthesis kits (Invitrogen). Normalized RNA and cDNA were stored at −80°C between steps.

Gene expression was quantified using the assays and samples in singleton with specific target amplification (STA) enriched cDNA and the Fluidigm platform as described above. We included additional assays for candidate genes of thermal and hypoxic stress to assess cross-reactivity with candidate smoltification genes (data available from authors). Each gene expression chip contained three housekeeping genes (i.e. Coil-P84, 78d16.1 and MrpL40; Miller *et al*., 2017), dilutions of a group-specific cDNA pool and a group-specific calibrator sample. For determining the optimal normalization gene(s) from the three housekeeping (HK) candidates, gene expression of each HK was first linearly transformed (efficiency ^minimum Ct−sample Ct^). Values were then used in the NormFinder R function (Andersen *et al*., 2004) with groupings for constituents (e.g. hatchery location) by month to identify the gene or gene pair with the best stability (lowest standard deviation). Sample gene expression was normalized with the ∆∆Ct method (Livak and Schmittgen, 2001) using the mean (for single gene) or geometric mean (for pair of genes) and the group-specific calibrator sample. Gene expression was then log transformed: log_2_(2^-∆∆Ct^).

### Statistical analysis for validating genes

Candidate smoltification genes were validated using a correlation analysis based on principal components analyses (PCA) across groups and within groups. Analyses were performed using R 3.4.4 at a significance level of α = 0.05. Across the four groups, the expression values of all freshwater monthly gill samples were placed into a single PCA. Loadings and scores were visualized using the ‘fviz_pca’ function of the ‘factoextra’ R package (Kassambara and Mundt, 2017). The PC axis best separating earlier and later months was identified. Candidate genes were ranked as biomarkers based on the significance of Pearson correlations between each gene assay and this PC axis. A second PCA and visualization was performed using the top 10 biomarkers with *P* < 0.05. Additional Pearson correlations examined the relationships between gene expression patterns (PC1 and PC2 of the second PCA) and NKA activity, as well as body length, mass, condition, morphology and skin pigmentation. The same approach was used to examine each of the four groups separately. Student’s *t*-tests also examined gene expression differences for all 37 gene assays between freshwater and seawater samples collected at the same time in late April for Nitinat ocean-type Chinook salmon.

**Table 2:** Summary of the candidate smoltification biomarkers for qPCR assay design using gill tissue

Gene symbol	Gene name	Functional group	Probe ID	Gene ID	44K analysis	16K analysis	Literature mining
**Upregulated in smolt**							
CA4	Carbonic anhydrase 4	Ion regulation	C148R144	106569487	x		
CFTR-I	Cystic fibrosis transmembrane conductance regulator I	Ion regulation	C161R157	100136364	x		x
NKAa1-b	Na^+^/K^+^-ATPase α-1b (seawater)	Ion regulation	C230R144	100136390	x		x
NKCC	Na^+^/K^+^/2Cl^−^ co-transporter	Ion regulation	C188R143	112220018^a^	x		x
HBA	Hemoglobin subunit α	Oxygen transport	C228R104	106601077	x		x
HBAt	Hemoglobin subunit α (true HBA)	Oxygen transport	C109R104	100136572	x		x
RHAG	Rhesus blood group-associated glycoprotein	Oxygen transport	C069R106	100136438	x		
MPC1	Mitochondrial pyruvate carrier 1-like	Metabolic rate	C010R030	106612504	x		x
GAPDH	Glyceraldehyde−3−phosphate dehydrogenase	Metabolic rate	C146R081	106569991	x		x
NDUFB2	NADH dehydrogenase 1 beta subcomplex subunit 2	Metabolic rate	C037R160	106576359		x	x
NDUFB4	NADH dehydrogenase 1 beta subcomplex subunit 4	Metabolic rate	C216R021	100196139	x		
RPL31	60S ribosomal protein L31	Growth	C209R008	106582252	x		x
SLC16A10	Monocarboxylate transporter 10-like	Growth	C230R050	106571314	x		
EEF2	Elongation factor 2	Growth	-	100194965			x
CYP2K1	Cytochrome P450 2K1	Calcium uptake	C247R082	106572755	x		x
S100A4	Protein S100-A4	Calcium uptake	C153R120	100196458	x^b^		
WHRN	Whirlin	Structural integrity	C105R124	106585216	x		
ACTB	Beta actin	Structural integrity	-	100136352			x
TSPO	Translocator protein	Immunity	C213R123	100286416	x	x	
RGS5	Regulation of G protein signalling 5	Immunity	C212R121	106560296	x		
FKBP5	FK506-binding protein 5	Immunity	C148R059	106565346	x^c^		
CLEC4M	C-type lectin domain family 4 member M	Immunity	C010R062	106578890			x
THRB1	Thyroid hormone receptor beta 1	Hormone	C139R155	100136934			x
GHR1	Growth hormone receptor 1	Hormone	-	100136442			x
NR3C1	Glucocorticoid receptor 1	Hormone	-	100380779			x
**Downregulated in smolt**							
NKAa1-a	Na^+^/K^+^-ATPase α-1a (freshwater)	Ion regulation	C217R121	106610479			x
GlyT2	Na- and Cl-dependent glycine transporter 2	Ion regulation	C017R076	106561903	x		
CCL4	C-C motif chemokine 4	Immunity	C240R068	106585882	x		
CCL19	C-C motif chemokine 19	Immunity	C188R011	106585878	x		
IFI44	Interferon-induced protein 44	Immunity	C260R153	106573916	x		
MS4A4A	Membrane-spanning 4-domains A-4A	Immunity	C023R137	106605437	x		
PLK2	Serine/threonine-protein kinase PLK2	Immunity	C164R090	100195918	x		x
CD3Z	T-cell surface glycoprotein CD3 zeta chain precursor	Immunity	C241R010	106575734	x		
UBA1	Ubiquitin-like modifier-activating enzyme 1 X	Immunity	C230R063	106566065	x		
EXO1	Exonuclease 1	Immunity	C090R027	106576944	x		
NAMPT	Nicotinamide phosphoribosyltransferase	Immunity	C259R043	106561705	x		
IL12B	Interleukin-12 beta	Immunity	C095R005	106603888	x		
MCM4	DNA replication licensing factor MCM4-B	Immunity	C124R129	106569128	x	x	x
TUBA8L2	Tubulin, alpha 8 like 2	Immunity	C218R157	100194601		x	
FMNL1	Formin-like protein 1	Immunity	C217R022	106601135		x	
TRA	T-cell receptor alpha	Immunity	C123R016	106569062		x	
TYK2	Non-receptor tyrosine-protein kinase	Immunity	C058R025	106597276		x	
WAS	Wiskott–Aldrich Syndrome protein	Immunity	C052R071	106567248		x	x
RGS21	Regulator of G-protein signalling 21	Immunity	C097R005	106598526			x^d^
PRLR	Prolactin receptor	Hormone	-	100136497			x

Presented for each gene is the smoltification functional group, feature (probe) ID for the 44K cGRASP microarray and Atlantic salmon (*S. salar*) gene ID. The symbol x indicates that the gene was significant for parr to smolt for a specified analysis.

a ^a^Unknown Atlantic salmon gene ID for NKCC co-transporter, so the Chinook salmon gene ID is given.

b ^b^Protein S100-A4 was identified as highly significant by Sutherland *et al.* (2014), checked visually for Sockeye salmon dataset using boxplots.

c ^c^FK506-binding protein 5 was identified as highly by Sutherland *et al.* (2014), checked visually for Sockeye salmon dataset using boxplots.

d ^d^Regulator of G-protein signalling was identified by literature mining; subunit 21 was identified from Sockeye salmon dataset using boxplots.

### Seawater tolerance classification model

We conducted a companion study using juvenile ocean-type Chinook salmon exposed to salinity treatments (freshwater, brackish and seawater) during four trials that spanned the smoltification period (Houde *et al*., 2019). Each trial was categorized as pre-smolt, smolt or de-smolt based on fish survival several days after an acute seawater transfer of a subset of individuals. The PCA pattern for the ocean-type Chinook salmon in the present study was applied to the freshwater juveniles of the companion study. Gene expression PC axis thresholds that best separated the three smolt statuses then were identified by the maximum of Youden’s J statistic (sensitivity + specificity −1, Youden, 1950) from receiver operating characteristic (ROC) analysis using the *pROC* R package. The resulting thresholds were used to classify fish as seawater tolerant (smolt) or intolerant (pre-smolt and de-smolt). By applying this seawater tolerance classification model to the ocean-type Chinook salmon we examined how seawater tolerance progressed on a monthly basis.

## Results

### Candidate smoltification genes

A total of 45 candidate smoltification genes were selected for TaqMan qPCR design: 25 upregulated and 20 downregulated for parr to smolt (Table 2). The majority of the candidate genes (*n* = 34) were from the microarray analyses using both Sockeye salmon and Rainbow trout; 13 of these genes were also present in the literature review (see Supplementary Methods). Of the 34 genes, 28 were from the 44K analysis and mainly represented the extremes of the fold changes and six were from the 16K analysis and represented most of the available genes for this analysis. Another two genes (S100A4 and FKBP5; gene symbols described in Table 2) were identified as highly differentially expressed by Sutherland *et al*. (2014) for Rainbow trout and were added by visual inspection of Sockeye salmon boxplots. The last nine genes were from the literature mining to fill eight biological functions, i.e. ion regulation, oxygen transport, metabolic rate, growth, calcium uptake, structural integrity, immunity and hormones, so there would be at least two representative genes. A total of 8 out of 45 assays did not pass the efficiency criteria (i.e. CD3Z, GAPDH, GlyT2, NKCC, RGS5, TYK2, S100A4 and WHRN) across species (see Supplementary Methods), thus leaving 20 upregulated gene assays and 17 downregulated genes.

**Figure 1:**
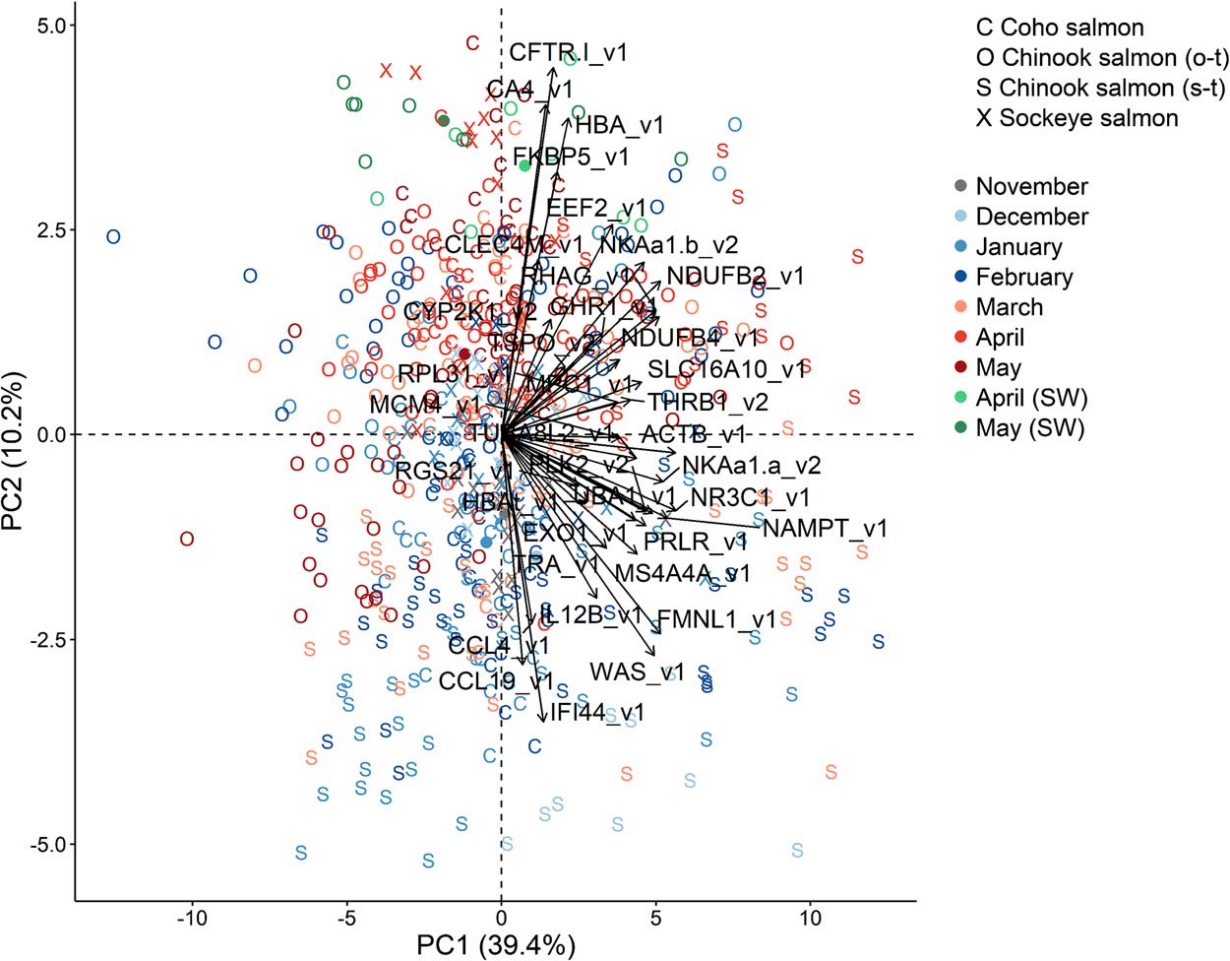
Plots of the first two principal components of all 37 candidate genes for smoltification using all four groups. Groups are Coho salmon (*O. kisutch*), Sockeye salmon (*O. nerka*), Chinook salmon (stream-type, *O. tshawytscha*) and Chinook salmon (ocean-type, *O. tshawytscha*). Percentage in brackets is the variation explained by the component. Monthly sample centroids are represented by the circle of the same colour. Black arrows represent loading vectors of the biomarkers. Legend symbol SW is for seawater and these individuals were not used in the PCA.

### Validation of smoltification genes

**Figure 2:**
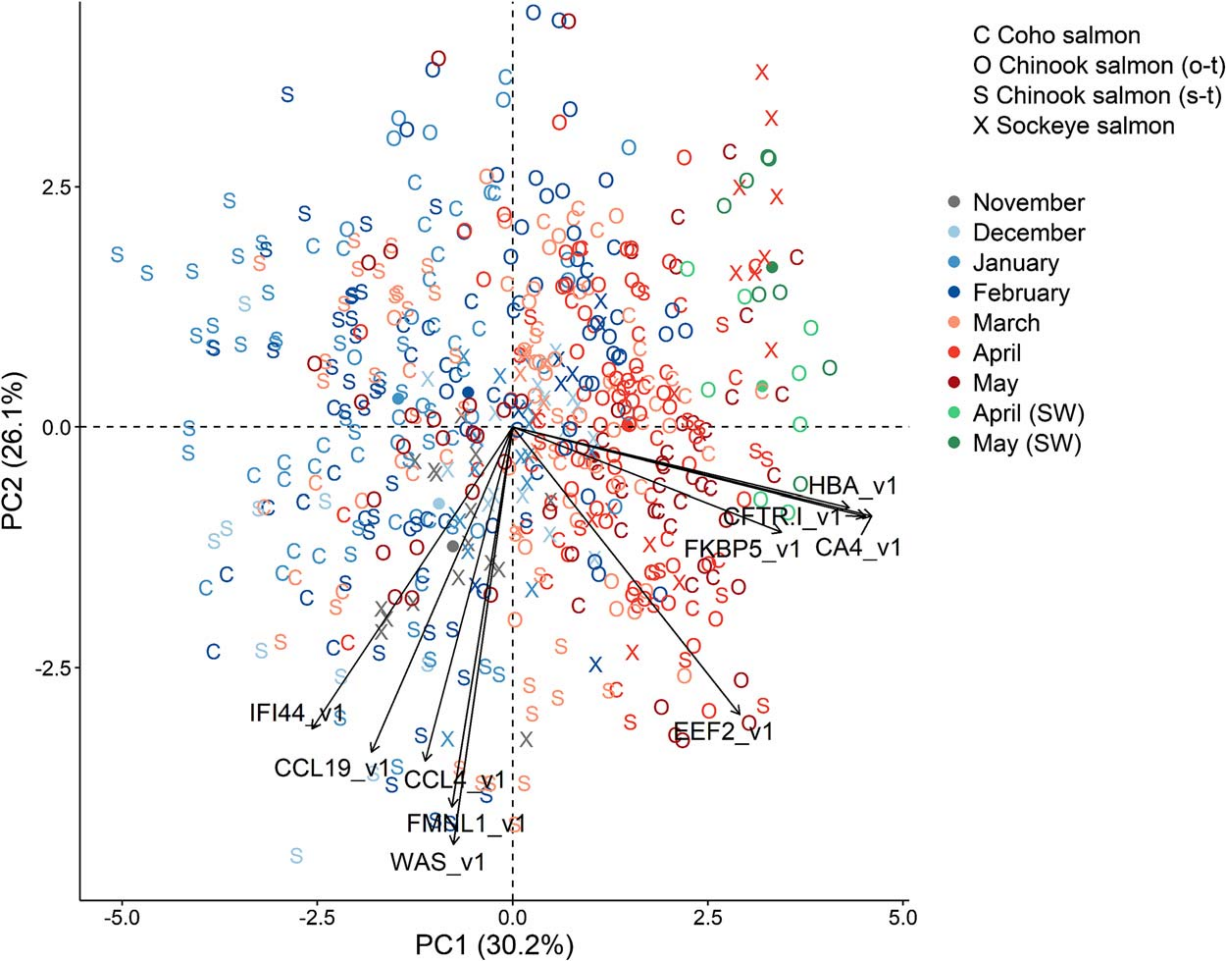
Plots of the first two principal components of the top 10 biomarkers for smoltification using all four groups. Groups are Coho salmon, Sockeye salmon, stream-type Chinook salmon and ocean-type Chinook salmon. See Fig. 1 legend.

Across all four groups, a PCA of gill expression of 37 candidate genes identified that PC2 separated earlier and later months (Fig. 1). PC1 was associated with group differences. The expression of 32 genes was significantly correlated (*P* < 0.05) with PC2 (summary in Table 3; see Supplementary Analysis). The top 10 genes based on correlation significance were represented by five upregulated biomarkers and five downregulated biomarkers in smolts (Fig. 2). Gene expression values for all four groups are provided in the Supplementary Data.

**Figure 3:**
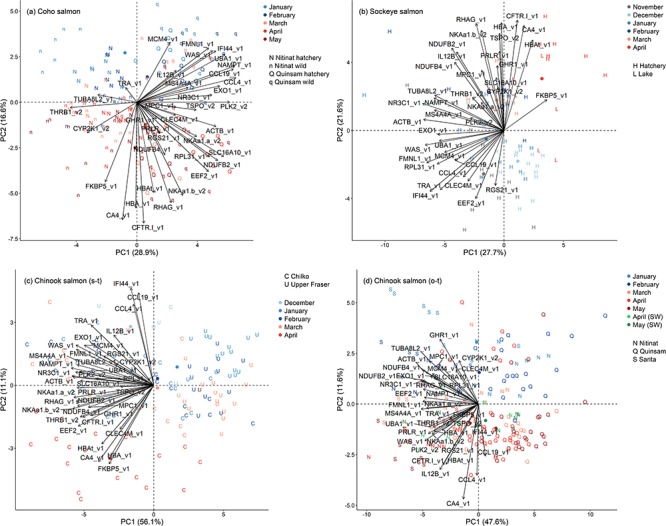
Plots of the first two principal components of all 37 candidate genes for smoltification using each of the four groups. (a) Coho salmon, (b) Sockeye salmon, (c) stream-type Chinook salmon and (d) ocean-type Chinook salmon. See Fig. 1 legend.

**Figure 4:**
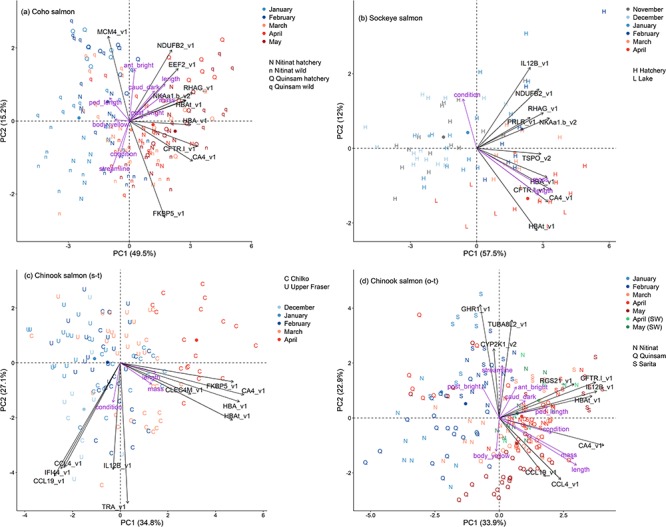
Plots of the first two principal components of the top 10 biomarkers for smoltification using each of the four groups. (a) Coho salmon, (b) Sockeye salmon, (c) stream-type Chinook salmon and (d) ocean-type Chinook salmon. Purple arrows represent loading vectors of the body variables. See Fig. 1 legend.

Within each of the four groups, PCAs of gill expression of 37 candidate genes identified that PC2 separated earlier and later months (Fig. 3). PC1 was associated with different sets, i.e. hatchery or wild and source population. Coho salmon had 26 genes, Sockeye salmon had 28 genes, stream-type Chinook salmon 21 genes and ocean-type Chinook salmon had 30 genes with expression values significantly correlated with PC2 (Table 3). Notably, ocean-type Chinook salmon had metabolic rate and growth genes downregulated, and eight immunity genes upregulated during smoltification, opposite the prediction. Five biomarkers, i.e. CA4, CFTR-I, HBA, HBAt and NKAa1b (gene symbols described in Table 2), were consistently upregulated across all groups (Fig. 4). An additional four biomarkers, i.e. CCL19, CCL4, IFI44 and IL12B, were consistently downregulated for Coho salmon, Sockeye salmon and stream-type Chinook salmon, but upregulated for ocean-type Chinook salmon.

Comparing Nitinat ocean-type Chinook salmon collected at the same time in late April from freshwater and seawater (about 2 weeks exposure to an estuary), 13 of the 30 genes were differently expressed between environments (Table 3; see Supplementary Analysis). Interestingly, the genes predicted to be downregulated during smoltification were first upregulated in freshwater and only downregulated in seawater.

### Relationship to gill NKA activity and body variables

**Figure 5:**
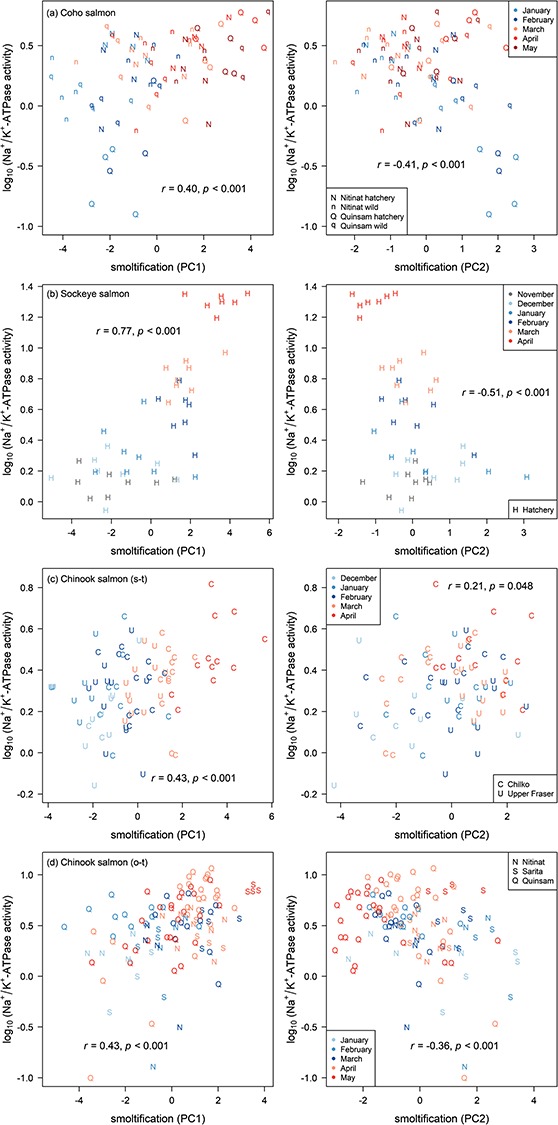
Relationships between smoltification gene expression patterns and NKA activity for the four groups. By row: (a) Coho salmon, (b) Sockeye salmon, (c) stream-type Chinook salmon and (d) ocean-type Chinook salmon. Gene expression patterns used the top 10 biomarkers. Gill NKA activity units are μmol ADP (mg protein)^−1^ h^−1^, which are presented as log_10_. There were no samples for Sockeye salmon from Cultus Lake in April. Legend symbol SW is for seawater.

Smoltification biomarker panels for each of the four groups, i.e. PC1 and PC2 using the top 10 genes (Fig. 4), were significantly correlated with gill NKA activity (Fig. 5). Body length and mass were positively correlated with PC1 for each of the four groups, as expected for juveniles growing during smoltification (Fig. 4; see Supplementary Analysis). Body condition was also correlated with PC1 for ocean-type Chinook salmon, whereas it was correlated with PC2 for Coho salmon, Sockeye salmon and stream-type Chinook salmon. Gill NKA activity and body size values for all four groups are provided in the Supplementary Data.

Photographs to examine for correlations with skin pigmentation and body morphology were available only for the Coho salmon and ocean-type Chinook salmon. We considered the first four principal component axes (PCs) for skin pigmentation and two relative warps axes (RWs) for body morphology. For skin pigmentation, Coho salmon and Chinook salmon PC1 (53.8% and 37.3%) and PC2 (23.1% and 31.2%) were primarily associated with the posterior and anterior region brightness, respectively. Coho salmon PC3 (10.1%) was associated with body (posterior, anterior and caudal fin) region yellowness and PC4 (5.7%) with caudal fin darkness; these traits were PC4 (6.5%) and PC3 (19.3%) for Chinook salmon, respectively. For body morphology, we considered the RWs for truncated to streamlined body shape, i.e. Coho salmon RW2 (12.6%) and Chinook salmon RW5 (6.6%) and caudal peduncle length, i.e. RW7 (4.5 and 3.9%), because of their relationship with smoltification (McCormick *et al*., 1998, 2013; Björnsson *et al*., 2011).

The smoltification biomarker PC1s for both groups were positively correlated with caudal fin darkness (Fig. 4; see Supplementary Analysis). Coho salmon PC1 also had a positive trend for posterior brightness, as well as negative correlations with streamlined to truncated shape and caudal peduncle length and there was a trend for body yellowness. PC2 was correlated with anterior brightness. Chinook salmon PC1 was also positively correlated with caudal peduncle length. PC2 was correlated with posterior brightness, anterior brightness, body yellowness and streamlined to truncated shape. Skin pigment and body morphology values for all four groups are provided in the Supplementary Data.

**Table 3:** Summary of the gill smoltification gene expression patterns for the four groups

Gene name	Assay name	All groups	Coho salmon	Sockeye salmon	Chinook salmon (s-t)	Chinook salmon (o-t)	Seawater
**Upregulated in smolt (predicted)**							
Beta actin	ACTB_v1		+			−	
Carbonic anhydrase 4	CA4_v1	+	+	+	+	+	+
Cystic fibrosis transmembrane conductance regulator I	CFTR.I_v1	+	+	+	+	+	+ (t)
C-type lectin domain family 4 member M	CLEC4M_v1	+		−	+	−	
Cytochrome P450 2 K1	CYP2K1_v2	+	+	+	- (t)	−	
Elongation factor 2	EEF2_v1	+	+	−	+	- (t)	
FK506-binding protein 5	FKBP5_v1	+	+	+	+	+ (t)	
Growth hormone receptor 1	GHR1_v1	+		+	+	−	
Hemoglobin subunit α	HBA_v1	+	+	+	+	+	
Hemoglobin subunit α (true)	HBAt_v1	−	+	+	+	+	
Mitochondrial pyruvate carrier 1	MPC1_v1	+		+	+ (t)	−	
NADH dehydrogenase 1 beta subcomplex subunit 2	NDUFB2_v1	+	+	+		−	−
NADH dehydrogenase 1 beta subcomplex subunit 4	NDUFB4_v1	+	+	+	+ (t)	−	
Na^+^/K^+^-ATPase α-1b (seawater)	NKAa1.b_v2	+	+	+	+	+	
Glucorticoid receptor 1	NR3C1_v1	−		+ (t)		−	
Rhesus blood group-associated glycoprotein	RHAG_v1	+	+	+		−	−
60S ribosomal protein L31	RPL31_v1		+	−		−	
Monocarboxylate transporter 10	SLC16A10_v1	+	+	+		−	
Thyroid hormone receptor beta 1	THRB1_v2			−	+	+	- (t)
Translocator protein	TSPO_v2	+		+		+	+
**Downregulated in smolt (predicted)**							
C-C motif chemokine 19	CCL19_v1	−	−	−	−	+	−
C-C motif chemokine 4	CCL4_v1	−	−	−	−	+	−
Exonuclease 1	EXO1_v1	−			−	−	
Formin-like protein 1	FMNL1_v1	−	−	−	−		
Interferon-induced protein 44	IFI44_v1	−	−	−	−	+	−
Interleukin-12 beta	IL12B_v1	−	−	−	−	+	- (t)
DNA replication licensing factor MCM4	MCM4_v1		−		−	−	
Membrane-spanning 4-domains A-4A	MS4A4A_v1	−			−		- (t)
Nicotinamide phosphoribosyltransferase	NAMPT_v1	−	−	−			−
Na^+^/K^+^-ATPase α-1a (freshwater)	NKAa1.a_v2	−	+	+ (t)			−
Serine/threonine-protein kinase PLK2	PLK2_v2	−				+	
Prolactin receptor	PRLR_v1	−	+	+		+	−
Regulator of G-protein signalling 21	RGS21_v1	−	+	−	−	+	+
T-cell receptor alpha	TRA_v1	−		−	−	+ (t)	
Tubulin, alpha 8 like 2	TUBA8L2_v1			−	- (t)	−	
Ubiquitin-like modifier-activating enzyme 1 X	UBA1_v1	−	−			+	−
Wiskott–Aldrich Syndrome protein	WAS_v1	−	−		−	+	−

The expression values of the 37 candidate genes were subjected to PCA for all four groups and each group separately: Coho salmon, Sockeye salmon, stream-type Chinook salmon and ocean-type Chinook salmon. Gene expression relationships with the main PC axis separating earlier and later months were examined. Student *t*-tests examined expression differences between freshwater and seawater Nitinat ocean-type Chinook salmon sampled at the same time in late April; estuary juveniles were exposed to seawater for about 2 weeks. Presented are the significant (*P* < 0.05) expression patterns: + for positive correlation with smoltification or higher in seawater and − for negative correlation with smoltification or lower in seawater. Trends (*P* < 0.1) are presented with a t in brackets. Pearson correlations and *P*-values, as well as freshwater and seawater mean differences and statistics, are displayed in the Supplementary Analysis.

### Seawater tolerance classification model

**Figure 6:**
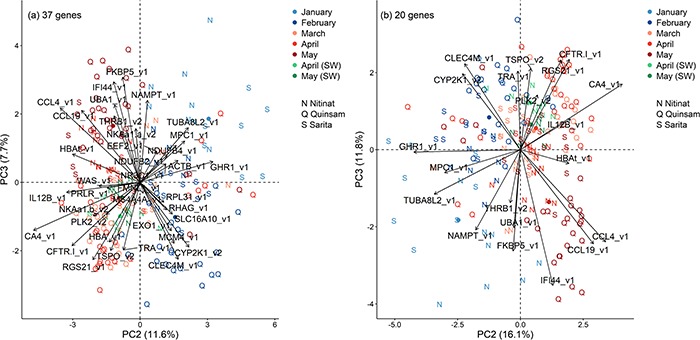
Plots of the second and third principal components for the candidate genes using ocean-type Chinook salmon. Displayed are (a) all 37 genes and (b) the top 20 genes. See Fig. 1 legend.

The initial PCA of the gill expression using 37 candidate genes for ocean-type Chinook salmon in the present study indicated a pre-smolt to smolt pattern for PC2 and suggested a smolt to de-smolt pattern for PC3 (Fig. 6a). Specifically, Quinsam May juveniles separated from earlier months along PC3. De-smoltification was also suspected for Quinsam May juveniles because of a decrease in gill NKA activity (mean ± SE, April 5.7 ± 0.7 and May 3.9 ± 0.4 μmol ADP (mg protein)^−1^ h^−1^, Student’s *t*-test *P* = 0.028). PC3 was significantly correlated with the expression of 25 genes (see Supplementary Analysis).

**Figure 7:**
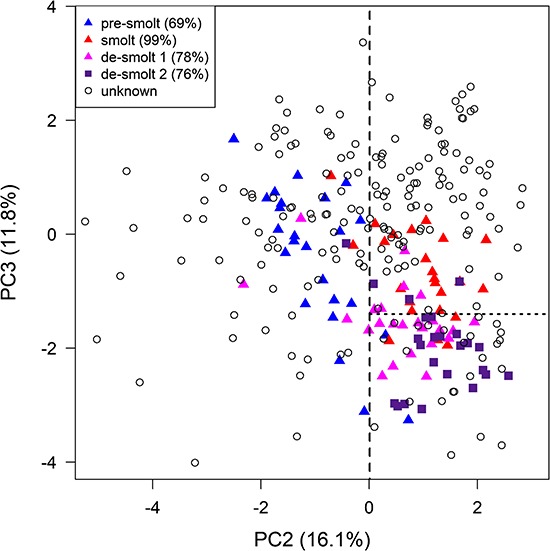
Seawater tolerance classification model using gene expression patterns of ocean-type Chinook salmon. Freshwater individuals with a smolt status are from the four trials of the companion study of Houde *et al*. (2019). Percentages for smolt statuses represent the trial seawater survival. The plot is based on the PCA using the top 20 biomarkers displayed in Fig. 6b, and individuals of the companion study were projected into PC2 and PC3. Dashed lines represent the PC axis thresholds that separate (i) pre-smolt and smolt and (ii) smolt and de-smolt. Thresholds were determined using Youden’s J statistic and ROC analysis. Juveniles within the ‘smolt’ area were classified as seawater tolerant and juveniles within the ‘pre-smolt’ and ‘de-smolt’ areas were classified as not seawater tolerant.

A new PCA using the top 20 biomarkers (*P* < 1 × 10^−5^ for both PC2 and PC3) maintained patterns as expected (Fig.6b), and the freshwater individuals of a companion study (Houde *et al*., 2019) were projected into this PCA. These freshwater individuals were assigned a smolt status at the trial level based on the survival (over several days) of other individuals from the same trial during acute seawater transfer. The best PC2 threshold separating pre-smolt and smolt trials (maximum of Youden’s J statistic and ROC analysis) was 0.01, and the best PC3 threshold separating smolt and de-smolt trials was −1.40 (Fig. 7). Individuals were classified as seawater tolerant (smolt) or intolerant (pre-smolt and de-smolt) using the areas defined by the thresholds.

The classification model was applied to the unknown smolt status ocean-type Chinook salmon of the present study. Nitinat and Sarita juveniles were largely classed as seawater intolerant pre-smolt from January to March and seawater tolerant smolt in April and May (Table 4). On the other hand, Quinsam juveniles were classed as seawater intolerant pre-smolts in February, seawater tolerant smolts in March and April and largely seawater intolerant de-smolts in May.

## Discussion

Comparing gill gene expression for anadromous Rainbow trout (Sutherland *et al*., 2014) with our internal Sockeye salmon dataset, we discovered numerous common candidate smoltification genes. Specifically, a subset of 25 upregulated and 20 downregulated genes were selected for TaqMan qPCR assay design. Of these 45, which mainly represented the fold change extremes of the 44K analysis, 20 upregulated and 17 downregulated genes passed our assay efficiency criteria and then were applied to our monthly gill analysis of Coho salmon, Sockeye salmon, stream-type Chinook salmon and ocean-type Chinook salmon. While 32 common smoltification biomarkers were identified, smoltification biomarkers ranged from 21 to 30 genes within each group. Nevertheless, smoltification biomarkers regardless of grouping could be reduced to a top 10 genes while retaining good separation along the smoltification axis. Indeed, smoltification gene expression patterns (i.e. PC1 and PC2 of the biomarker panels using the top 10 genes for each group) were confirmed by correlations with gill NKA activity. Thus, we recommend these top 10 genes for smoltification biomarkers panels of the four groups (Fig. 4). For species and ecotypes not examined in the present study, e.g. Atlantic salmon, we recommend the smoltification biomarker panel using the top 10 genes for the groups combined (Fig. 2).

### Common gill smoltification genes among groups

Across the four groups, smoltification triggered upregulation of ion regulation (carbonic anhydrase 4, CA4; cystic fibrosis transmembrane conductance regulator I, CFTR-I; and Na^+^/K^+^-ATPase α-1b, NKAa1-b) and oxygen transport (hemoglobin alpha, HBAt and HBA) genes. Another oxygen transport gene (Rhesus blood group-associated glycoprotein, RHAG) was also upregulated for Coho salmon and Sockeye salmon. CFTR-I and NKA1a-b are important ion regulators for gill ionocytes that help remove excess chloride and sodium ions for fish in seawater (Evans *et al*., 2005).

Furthermore, four immunity genes (C-C motif chemokine 19, CCL19; C-C motif chemokine 4, CCL4; interferon-induced protein 44, IFI44; and interleukin-12 beta, IL12B) were downregulated during smoltification for Coho salmon, Sockeye salmon and stream-type Chinook salmon, but upregulated for ocean-type Chinook salmon (elaborated below). Yet, these four genes had lower expression in seawater than freshwater for ocean-type Chinook salmon. The majority of immunity genes (300 out of 360), such as chemokines, can be downregulated during seawater acclimation, possibly because of a trade-off between the energetic costs of osmoregulation and pathogen resistance in seawater (Johansson *et al*., 2016). These eight genes were predominantly at the top end of upregulated and downregulated genes (based on fold change) in the 44K analysis, but importantly were not detected in the 16K analysis. The upregulated genes and chemokines were also identified by literature mining. Four uncharacterized features showed downregulation in the 44K analysis, but limited sequence template precluded assay design. They may be worth pursuing should more sequence data become available.

The consistency of these ion regulator genes across groups suggests that the Na^+^/K^+^/2Cl^−^ cotransporter (NKCC), also within gill ionocytes, may also be a good species-wide smoltification biomarker (see Nilsen *et al*., 2007; Stefansson *et al*., 2007). Unfortunately, our single assay for NKCC only worked for Rainbow trout, possibly because at the time we had limited sequence information; thus, we were not able to examine this gene for our target salmonids. Relative to the other ion regulators, carbonic anhydrase has received lesser research attention. Yet recently, carbonic anhydrase genes were under rapid genetic selection for osmoregulation of Rainbow trout introduced from high to low salinities (Willoughby *et al*., 2018). Carbonic anhydrase can be important for both acid-base and ion regulation because of the productions of H^+^ and HCO_3_^−^ needed for Na^+^ and Cl^−^ exchange in gill tissue (Gilmour, 2012; Havird *et al*., 2013). CA4 was the second most powerful single predictor of smoltification after CFTR-I using all groups.

Red blood cell hemoglobin isoforms change from juvenile to adult types during smoltification of Coho salmon and Sockeye salmon (Vanstone *et al*., 1964). The adult type may have a higher oxygen affinity and weaker Bohr effect than the juvenile type, suggesting an adaptation to the lower oxygen tension of seawater than freshwater. Yet, Fyhn *et al*. (1991) found that the isoforms shifted after smoltification for stream- and ocean-type Chinook salmon, suggesting that they may be more body size dependent. However, our findings of changes in hemoglobin genes during smoltification for stream- and ocean-type Chinook salmon suggest an importance of hemoglobin for smoltification in Chinook salmon, but not necessarily related to isoform switching.

Our confidence in the identified smoltification gene expression biomarkers is strengthened by the similarities in the response to higher salinity. A companion study used these same assays on juvenile ocean-type Chinook salmon exposed to freshwater (0 PSU), brackish (20 PSU) and seawater (28 or 29 PSU) for 6 days (Houde *et al*., 2019). Ion regulation genes (i.e. CA4, CFTR-I and NKAa1-b) and an oxygen transport gene (i.e. HBA) were upregulated in brackish and seawater than freshwater, as during smoltification in the present study. Similarly, the four immunity genes (i.e. CCL19, CCL4, IFI44 and IL12B) had a lower expression in brackish and seawater than freshwater and were downregulated during smoltification in the present study. Overall, we propose that, across all four Pacific salmonid groups examined, the strongest and most consistent smoltification biomarkers were those required for the higher salinity and lower oxygen tension in seawater relative to freshwater.

### Different gill smoltification genes among groups

Beyond ion regulation and oxygen transport, gene expression patterns for the remaining six upregulated biological functions were dependent on the group or did not fit the prediction based microarray or literature information. In particular, three metabolic rate genes (NADH dehydrogenase 1 beta subcomplex subunit 2 and 4, NDUFB2 and NDUFB4, and mitochondrial pyruvate carrier 1, MPC1) were generally upregulated for Coho salmon, Sockeye salmon and stream-type Chinook salmon, but downregulated for ocean-type Chinook salmon. Expression of metabolic rate genes can be related to body growth (Salem *et al*., 2007), and importantly photoperiod is known to influence growth of stream- but not ocean-type Chinook salmon (Clarke *et al*., 1992, 1994). Conceivably, metabolic rate gene expression may differ as a result of photoperiod dependence, but a mechanistic link would need to be found.

**Table 4:** Modelled seawater tolerance by monthly development for ocean-type Chinook salmon

Month	Seawater tolerance		
	Pre-smolt	Smolt	De-smolt
Nitinat			
Jan	6	0	2
Feb	8	0	0
Mar	7	1	0
Apr	1	14	1
Sarita			
Jan	8	0	0
Feb	8	0	0
Mar	4	4	0
Apr	2	6	0
May	2	5	1
Quinsam			
Feb	18	4	0
Mar	0	14	0
Apr	6	22	0
May	4	2	20

The classification model used gene expression pattern thresholds for delineating seawater tolerant (smolt) and intolerant (pre-smolt and de-smolt). Month symbols are in chronological order of development and are the first three letters.

Three growth genes (monocarboxylate transporter 10, SLC16A10; elongation factor 2, EEF2; and 60S ribosomal protein L31, RPL31) were also generally upregulated for Coho salmon or stream-type Chinook salmon, but downregulated for Sockeye salmon or ocean-type Chinook salmon even though these two groups also continued to grow. Thus, elongation factors and ribosomal genes may not be consistently upregulated during smoltification, e.g. downregulation of elongation factor 1B and upregulation of ribosomal proteins (Lemmetyinen *et al*., 2013), downregulation of ribosomal proteins (Seear *et al*., 2010) and mixture of up and downregulation of ribosomal proteins (Boulet *et al*., 2012; Robertson and McCormick 2012).

The structural integrity gene (beta actin, ACTB) did not change with smoltification for Sockeye salmon and stream-type Chinook salmon. Hecht *et al*. (2014) also found no change with ACTB for Rainbow trout. The calcium uptake gene (cytochrome P450 2K1, CYP2K1) was upregulated for Coho salmon and Sockeye salmon, but downregulated for stream- and ocean-type Chinook salmon. Another calcium uptake gene, protein S100-A4 (S100A4), had the largest parr-to-smolt difference in expression for the Rainbow trout microarray study (Sutherland *et al*., 2014); unfortunately, our assay for S100A4 did not work for Chinook salmon and Sockeye salmon, so this gene was not examined further. One gene each represented the structural integrity and calcium uptake biological functions. Future work should examine other structural integrity genes such as collagen, SPARC or tropomyosin (e.g. Seear *et al*., 2010; Lemmetyinen *et al*., 2013) and develop an assay for S100A4 that works on a broader range of species to examine the consistency of regulation across species and ecotypes.

Support was lacking across groups for any of the hormone genes and for two of three immunity genes predicted to be upregulated during smoltification. The immunity gene FK506-binding protein 5 was upregulated for Coho salmon, Sockeye salmon, stream-type Chinook salmon, with a similar trend for ocean-type Chinook salmon. On the other hand, translocator protein (TSPO) was upregulated for Sockeye salmon and ocean-type Chinook salmon only, and c-type lectin domain family 4 member M (CLEC4M) was upregulated for stream-type Chinook salmon only. In contrast to the Sockeye salmon and ocean-type Chinook salmon examined in the present study, Atlantic salmon, Rainbow Trout and Brook trout in previous studies (Seear *et al*., 2010; Boulet *et al*., 2012; Lemmetyinen *et al*., 2013; Sutherland *et al*., 2014) showed upregulation of c-type lectins 2 or 4M. Growth hormone receptor 1 (GHR1) was upregulated for Sockeye salmon and stream-type Chinook salmon but downregulated for ocean-type Chinook salmon. Glucocorticoid (cortisol) receptor 1 (NR3C1) was upregulated for Sockeye salmon (trend) but downregulated for stream-type Chinook salmon. Thyroid hormone receptor beta 1 (THRB1) was upregulated for both types of Chinook salmon only. Although plasma values of these hormones are associated with smoltification across species (e.g. McCormick *et al*., 2013), our results confirm previous studies suggesting that the gene expression patterns of these hormones or their receptors are not necessarily in line with plasma patterns (e.g. Kiilerich *et al*., 2007; Stefansson *et al*., 2007; Hecht *et al*., 2014). Overall, the immunity and hormone gene expression patterns suggest that there are species and ecotype differences during smoltification or that these genes are functioning outside of the smoltification process. Further studies should examine the reproducibility of these patterns across species and ecotypes.

Beyond the four immunity genes identified as being generally downregulated during smoltification, predicted downregulation of remaining gill genes was group depended. Immunity genes appear to be downregulated during smoltification for certain species and ecotypes, e.g. Sockeye salmon, while other species and ecotypes may not have a downregulation of these genes until reaching higher salinity, e.g. ocean-type Chinook salmon. Similar to Houde *et al*. (2019), Na^+^/K^+^-ATPase α-1a (NKAa1-a) and prolactin receptor (PRLR) were lower in seawater than freshwater for ocean-type Chinook salmon, and a higher expression of both genes was previously associated with mortality in seawater. Thus, there is further support for the suggestion that expression of these two genes should decrease for proper seawater acclimation (also see Flores and Shrimpton, 2012).

### Relationship to gill NKA activity

Elevated gill NKA activity is associated with survival in seawater of Atlantic salmon (e.g. Stich *et al*., 2015; Stich *et al*., 2016) and ocean-type Chinook salmon (Houde *et al*., 2019), as well as reduced risk of predation for Rainbow trout (Kennedy *et al*., 2007). Similarly, we found correlations between NKA activity and the primary smoltification gene expression pattern (PC1) across all four groups. The correlation is likely stronger for Sockeye salmon by using a 44K candidate gene discovery analysis. Although only moderate correlations are common between gene expression and protein activity (Schwänhausser *et al*., 2011; Kanerva *et al*., 2014), possibly because of post-transcriptional and post-translational modifications (Maier *et al*., 2009), changes in gene expression may be one of the first indicators of a physiological change or response (Feder and Walser, 2005; Miller *et al*., 2017). Furthermore, a high NKA activity preceding seawater entry may not be essential provided juvenile salmon can rapidly increase NKA activity once in seawater (Madsen and Naamansen, 1989; Bassett *et al*., 2018), as shown for Pink salmon (Sackville *et al*., 2012). Indeed, ocean-type Chinook salmon smolts in seawater had a higher median NKA activity than either pre-smolts or de-smolts (i.e. 10.2 vs. <7.5 μmol ADP (mg protein)^−1^ h^−1^; Houde *et al*., 2019).

### Relationship to body appearance

Gill gene expression patterns were clearly associated with skin pigmentation (i.e. body brightness and caudal fin darkness) and body morphology (i.e. caudal peduncle length), which are classical changes associated with smoltification. For example, lower body condition, more streamlined body shape, elongation of caudal peduncle, increased body silvering and darkening of caudal fin margins are commonly used smoltification indices (Carey and McCormick, 1998; Björnsson *et al*., 2011; McCormick *et al*., 2013). As far as we are aware, ours is the first study to relate gene expression patterns and body appearance during smoltification. Conceivably, caudal fin darkness may be a proxy of smoltification across other species and ecotypes but we did not have photographs of stream-type Chinook salmon and Sockeye salmon to test this possibility. Further research should examine whether these patterns occur in additional species and ecotypes.

### Seawater tolerance model

Our preliminary seawater tolerance classification model for ocean-type Chinook salmon incorporated the gene expression patterns of freshwater pre-smolt, smolt and de-smolt trials from a companion study using acute seawater transfers (Houde *et al*., 2019). Similar to Di Cicco *et al*. (2018) who classified viral disease states, we statistically identified the gene expression (PC2 and PC3) thresholds that best separated pre-smolt, smolt and de-smolt trials to classify individuals as seawater tolerant (smolt) or intolerant (pre-smolt and de-smolt). Our preliminary model appears to detect the gain as well as the loss of seawater tolerance using smolt status. Nitinat and Sarita juveniles were seawater tolerant in April and/or May around the hatchery release times, while Quinsam juveniles were seawater tolerant in March and April but were seawater intolerant (de-smolt) around the release times in May. The de-smoltification of May Quinsam juveniles was also confirmed by lower NKA activity. Even so, our discovery process for the candidate genes focused on smoltification, i.e. pre-smolt to smolt. Other genes (e.g. FKBP5, IFI44, NAMPT and UBA1), more frequent sampling and a longer sampling period into the summer may improve resolution between smolts and de-smolts.

Similar seawater tolerance classification models may be produced for Coho salmon, stream-type Chinook salmon and Sockeye salmon. Our preliminary model for ocean-type Chinook salmon used the freshwater smolt status at the level of the trial, with other individuals acutely transferred to seawater for measures of survival (Houde *et al*., 2019). A more direct approach of linking freshwater gene expression to seawater survival at the level of the individual would have been more powerful, for example, a small gill biopsy a few days before seawater transfer followed by a survival measure covering a few days after transfer, e.g. 6 days (Houde *et al*., 2019). Additional data are needed between individual gene expression and subsequent seawater tolerance to improve the model.

## Conclusion

Ion regulation, oxygen transport and certain immunity genes were consistently shown to be the best gill smoltification biomarkers across multiple population samples for four test groups. These identified genes were the top end of upregulated or downregulated genes based on fold changes, selected mainly from a 44K microarray discovery analysis. The directional shifts paralleled those previously seen with an experimental transition from freshwater to either brackish or seawater (Houde *et al*., 2019), implying higher salinity acclimation as being the trigger. Importantly, the identified smoltification gene expression patterns were significantly related to NKA activity and body indicators (caudal fin darkness for both Coho salmon and ocean-type Chinook salmon). Metabolic rate genes were upregulated and immunity genes were downregulated for photoperiod-dependent species and ecotypes such as stream-type Chinook salmon, Coho salmon and Sockeye salmon. However, the opposite occurred for photoperiod-independent species and ecotypes such as ocean-type Chinook salmon. Although we have clearly provided a preliminary classification system based on gene expression for seawater tolerance using pre-smolt, smolt and de-smolt ocean-type Chinook salmon, our classification system will need to be expanded to other species and ecotypes with individual-level data that link gene expression and seawater survival.

## Supplementary Material

suppl_methods_coz051

suppl_data_coz051

suppl_analysis_coz051
