# Supplementary material for: Discovery and validation of candidate smoltification gene expression biomarkers across multiple species and ecotypes of Pacific salmonids
Source: Conserv Physiol. 2019 Oct 11;7(1):coz051. doi: 10.1093/conphys/coz051 (PMC6788492; doi:10.1093/conphys/coz051)
Supplement: suppl_methods_coz051 [file suppl_methods_coz051.docx]

Supplemental Methods for **‘**Discovery and validation of candidate smoltification gene expression biomarkers across multiple species and ecotypes of Pacific salmonids’

*Candidate smoltification genes using microarray comparisons*

Limma analysis of the Sockeye salmon dataset identified 1,296 significant features that separated parr and smolt. The Rainbow trout dataset had a published 400 feature parr‒smolt signature. Combining the Sockeye salmon and Rainbow trout signatures using sIPCA to identify the top 100 features, there were 53 upregulated and 23 downregulated features that were also significant for the Sockeye salmon limma analysis (Table S1). Upregulated features were represented by six biological functions of metabolic rate (*n* = 21), immunity (8), oxygen transport (7), growth (7), structural integrity (5), ion regulation (4), and calcium uptake (1); the ion regulation and oxygen transport functions were predominantly at the top end of fold changes. Downregulated features were represented mainly by the function of immunity (17); other functions included ion regulation (1), growth (1), repressor of circadian rhythm (1), and unknown (3). Comparisons to three additional studies using the 16K platforms identified 7 upregulated and 14 downregulated significant features for parr to smolt for both species (Table S2). Upregulated features contained the functions of immunity (4), metabolic rate (2), and ion regulation (1). Downregulated features all contained the function of immunity.

*Candidate smoltification genes using literature mining*

Across the five published microarray studies, there were 15 upregulated and 6 downregulated genes for parr-to-smolt that were significant in at least two studies (Table S3). Including the studies that examined specific candidate genes, several of these studies found that the ion regulation genes Na^+^/K^+^-ATPase α-1 were significant, specifically for parr-to-smolt there was an upregulation of isoform ‘b’ and downregulation of isoform ‘a’ (e.g. Nilsen *et al.,* 2007; Piironen *et al.,* 2013; Stefansson *et al.,* 2007). There was also support for parr-to-smolt upregulation of other ion regulation genes, i.e. cystic fibrosis transmembrane conductance regulator I (e.g. Nilsen *et al.,* 2007) and Na^+^/K^+^/2Cl^-^ co-transporter (e.g. Nilsen *et al.,* 2007; Stefansson *et al.,* 2007). Although many plasma hormones change appreciably during smoltification (e.g. McCormick *et al.,* 2013), the majority of the associated genes were unchanged for parr-to-smolt in the five microarray studies. However, directed qPCR parr-to-smolt revealed significant upregulation of glucocorticoid (cortisol) receptor (Kiilerich *et al.,* 2007; but see Hecht *et al.,* 2014), growth hormone (Hecht *et al.,* 2014) and receptor (Hecht *et al.,* 2014; Kiilerich *et al.,* 2007; Stefansson *et al.,* 2007), insulin-like growth factor and receptor (Stefansson *et al.,* 2007), and thyroid receptor beta (Hecht *et al.,* 2014). Also, there may be a significant downregulation for parr-to-smolt of prolactin receptor (Kiilerich *et al.,* 2007; but see Hecht *et al.,* 2014). Hence, we included assays to these genes in our test panel.

*qPCR assay design*

A Microsoft Access relational database containing mRNA sequences of salmonids was produced for qPCR TaqMan assay design, with the objective of developing assays that were gene specific and worked across several species. The database is available from the authors. Until recently, there were limited mRNA sequence data for the genus *Oncorhynchus*, so sequences were generated for Coho (*O. kisutch*), Sockeye (*O. nerka*), and Chinook (*O.* *tshawytscha*) salmon from pools of six to eight individuals per species. Samples were enriched for GRASP microarray features using SureSelectXT (Agilent) and then sequenced using IonTorrent (Thermo-Fisher) following the manufacturer instructions. Akbarzadeh *et al.* (2018) provides greater methodological details.

Sequences were mapped to the Atlantic salmon genome using the methods described by Houde *et al.* (2019). The database also contained maps of microarray features to the Atlantic salmon genome, specifically GRASP (16K, 32K, and 44K), TRAITS (version 1, 2.1, and 2.2) and SIQ. Altogether, database retrieval of mRNA sequences for the three species used microarray features IDs, gene IDs, gene names, or official gene symbols. Available sequences of several salmonid species, i.e. from the database and additional searches of the NCBI repository, were aligned using MEGA7.0.14 (Kumar *et al.,* 2016). Sequences of closely related genes, e.g. duplicate genes, were also included in the alignment. Sequence regions that differed among closely related genes and were conserved among species were used as template in Primer Express 3.0.1 (Thermo Fisher) using the default setting optimized for the BioMark platform. Primer and TaqMan probe combinations that mismatched in at least one base pair at the 3’ end of closely related genes were preferentially selected. Another check of potential gene specificity and workability across salmonids used NCBI Primer-Blast. One or two assays were designed per candidate gene.

*Assay efficiencies*

Assay design efficiencies were measured using pools of cDNA samples for six to nine salmonid species. Species-specific pools contained a mix of five tissues (gill, liver, heart, kidney, and brain tissue) from several individuals, with the exception of Rainbow trout and Atlantic salmon (only gill tissue). For each species, cDNA was diluted using a five-fold serial dilutions 1 to 1/625. Following Fluidigm BioMark^TM^ prescribed methods, target cDNA sequences were enriched using a specific target amplification (STA) method that included small concentrations of the assay primers as well as three housekeeping genes: Coil-P84, 78d16.1, and MrpL40 (Miller *et al.,* 2017). Specifically, for each reaction, 3.76 μL 1X TaqMan PreAmp master mix (Applied Biosystems), 0.2 μM of each of the primers, and 1.24 μL of cDNA. Samples were run on a 14 cycle PCR program, with excess primers removed with EXO-SAP-IT (Affymetrix), and diluted 1 in 5 with DNA suspension buffer.

The sample dilutions were run in duplicate and most assays in singleton following the Fluidigm platform instructions. Specifically, for sample reactions, 3.0 μL 2X TaqMan mastermix (Life Technologies), 0.3 μL 20X GE sample loading reagent, and 2.7 μL STA product. For assay reactions, 3.3 μL 2X assay loading reagent, 0.7 μL DNA suspension buffer, 1.08 μL forward and reverse primers (50 uM), and 1.2 μL probe (10 uM). The PCR was 50°C for 2 min, 95°C for 10 min, followed by 40 cycles of 95°C for 15 s, and then 60°C for 1 min. Data were extracted using the Real-Time PCR Analysis Software (Fluidigm) using Ct thresholds set manually for each assay. Linear models of the Ct values by the log of the dilutions for each group-assay set were produced using the *plyr* R package (Wickham, 2011). Efficiency values were calculated using 10^-1/slope^ – 1, and assays with values between 0.9 and 1.1 were ideal.

For efficiency testing, two assays were designed for the top 12 upregulated and 10 downregulated genes (set 1), remaining genes had one assay design (set 2) (Table S4). Eight out of 45 assays did not pass the efficiency criteria (i.e. CD3Z, GAPDH, GlyT2, NKCC, RGS5, TYK2, S100A4, and WHRN) across species, thus leaving 20 upregulated gene assays and 17 downregulated genes (Table S5). For the genes with two assays (set 1), the assay with the highest efficiency across species was selected for further analysis with validation juvenile samples.

**References not in main text.**

Akbarzadeh A, Günther OP, Houde ALS, Ming TJ, Jeffries KM, Hinch SG, Miller KM (2018) Developing specific molecular biomarkers for thermal stress in salmonids. *BMC Genomics* 19:749.

Houde ALS, Schulze AD, Kaukinen KH, Strohm J, Patterson DA, Beacham TD, Farrell AP, Hinch SG, Miller KM (2019) Transcriptional shifts during juvenile Coho salmon (O*ncorhynchus kisutch)* life stage changes in freshwater and early marine environments. *Comp Biochem Phys D* 29:32-42.

Kumar S, Stecher G, Tamura K (2016) MEGA7: Molecular Evolutionary Genetics Analysis Version 7.0 for Bigger Datasets. *Mol Biol Evol* 33:1870-1874.

Wickham H (2011) The split-apply-combine strategy for data analysis. *J Stat Softw* 40:1-29.

Table S1. Summary of the results from the 44K analysis using the combined Sockeye salmon and Rainbow trout datasets for gill tissue.

The Rainbow trout dataset was from Sutherland et al. (2014). Sockeye salmon and Rainbow trout datasets were combined and analyzed collectively using a sparse independent principal component analysis to identify the top 100 features (i.e. Probe IDs) that separated parr and smolt. Presented are the 76 features that overlapped with the identified significant features of the separate Sockeye salmon robust limma analysis, which are ordered by fold change. Mean values are for parr and smolt of the Sockeye salmon dataset. Bold italics are gene names with Probe ID used for qPCR assay development; normal italics are gene names and Probe IDs matching to same gene ID.

| Probe ID | Gene symbol | Gene name | Functional group | Mean for parr | Mean for smolt | log_2_ fold change | Fold change | Adjusted *p-* value |
| --- | --- | --- | --- | --- | --- | --- | --- | --- |
| Upregulated in smolt | |  |  |  |  |  |  |  |
| ***C148R144*** | ***CA4*** | ***Carbonic anhydrase 4*** | ion regulation | -2.40 | 0.82 | 3.22 | 9.31 | 0.00 |
| ***C209R008*** | ***RPL31*** | ***60S ribosomal protein L31*** | growth | -1.04 | 1.08 | 2.12 | 4.34 | 0.01 |
| ***C228R104*** | ***HBA*** | ***Hemoglobin subunit alpha*** | oxygen transport | -1.66 | 0.32 | 1.98 | 3.96 | 0.00 |
| ***C105R124*** | ***WHRN*** | ***Whirlin*** | structural integrity | -0.84 | 0.97 | 1.81 | 3.51 | 0.00 |
| ***C069R106*** | ***RHAG*** | ***Rhesus blood group-associated glycoprotein*** | oxygen transport | -0.68 | 0.92 | 1.60 | 3.04 | 0.00 |
| ***C109R104*** | ***HBAt*** | ***Hemoglobin subunit alpha (true HBA)*** | oxygen transport | -1.25 | 0.30 | 1.55 | 2.93 | 0.03 |
| *C011R079* | *RHAG* | *Rhesus blood group-associated glycoprotein* | oxygen transport | -0.48 | 1.07 | 1.55 | 2.93 | 0.00 |
| ***C212R121*** | ***RGS5*** | ***Regulator of G-protein signalling 5*** | immunity | -0.93 | 0.48 | 1.41 | 2.65 | 0.03 |
| ***C161R157*** | ***CFTR-I*** | ***Cystic fibrosis transmembrane conductance regulator I*** | ion regulation | -1.65 | -0.39 | 1.26 | 2.40 | 0.01 |
| ***C230R144*** | ***NKAa1-b*** | ***Na^+^/K^+^-ATPase α-1b (seawater)*** | ion regulation | -1.22 | 0.04 | 1.26 | 2.39 | 0.01 |
| ***C213R123*** | ***TSPO*** | ***Translocator protein*** | immunity | -0.99 | 0.10 | 1.09 | 2.13 | 0.00 |
| C158R093 | RGS5 | Regulator of G-protein signaling 5 (gene ID 106584144) | immunity | -0.22 | 0.86 | 1.08 | 2.12 | 0.00 |
| ***C230R050*** | ***SLC16A10*** | ***Monocarboxylate transporter 10-like*** | growth | -0.17 | 0.91 | 1.08 | 2.11 | 0.00 |
| C146R037 | CDA | Cytidine deaminase | metabolic rate | -1.12 | -0.11 | 1.01 | 2.01 | 0.00 |
| ***C010R030*** | ***MPC1*** | ***Mitochondrial pyruvate carrier 1-like*** | metabolic rate | -0.44 | 0.55 | 0.98 | 1.98 | 0.04 |
| ***C247R082*** | ***CYP2K1*** | ***Cytochrome P450 2K1*** | calcium uptake | -0.40 | 0.51 | 0.91 | 1.88 | 0.04 |
| ***C188R143*** | ***NKCC*** | [***Na^+^/K^+^/2Cl^-^ co-transporter***](https://blast.ncbi.nlm.nih.gov/Blast.cgi#alnHdr_501418423) | ion regulation | -0.85 | 0.01 | 0.86 | 1.82 | 0.04 |
| C111R149 | SEC61G | Protein transport protein Sec61 subunit gamma | growth | -0.08 | 0.77 | 0.85 | 1.80 | 0.01 |
| C109R066 | TIMM17A | Mitochondrial import inner membrane translocase subunit Tim17-A | metabolic rate | -0.48 | 0.35 | 0.84 | 1.78 | 0.01 |
| ***C146R081*** | ***GAPDH*** | ***Glyceraldehyde-3-phosphate dehydrogenase*** | metabolic rate | -1.07 | -0.25 | 0.82 | 1.77 | 0.03 |
| C183R158 | BCKDK | 3-methyl-2-oxobutanoate dehydrogenase [lipoamide] kinase | metabolic rate | -0.61 | 0.19 | 0.80 | 1.74 | 0.01 |
| C005R080 | GLRX5 | Glutaredoxin-related protein 5 | oxygen transport | -0.52 | 0.24 | 0.77 | 1.70 | 0.02 |
| C069R043 | HIGD1A | HIG1 domain family member 1A | metabolic rate | 0.05 | 0.81 | 0.76 | 1.69 | 0.02 |
| C165R074 | RNF222 | RING finger protein 222 | structural integrity | -0.62 | 0.13 | 0.75 | 1.68 | 0.03 |
| C202R155 | At5g50100 | Uncharacterized protein At5g50100 | growth | -0.16 | 0.58 | 0.74 | 1.67 | 0.00 |
| C085R077 | CTNS | Cystinosin | immunity | -0.09 | 0.64 | 0.72 | 1.65 | 0.02 |
| C196R101 | PSPH | Phosphoserine phosphatase | metabolic rate | -0.46 | 0.24 | 0.70 | 1.63 | 0.04 |
| C221R144 | COX7A2 | Cytochrome c oxidase polypeptide VIIa-liver/heart | metabolic rate | -0.53 | 0.17 | 0.70 | 1.62 | 0.03 |
| C213R046 | PSPH | Phosphoserine phosphatase | metabolic rate | -0.42 | 0.27 | 0.69 | 1.62 | 0.01 |
| *C030R160* | *TSPO* | *Translocator protein* | immunity | -0.71 | -0.02 | 0.69 | 1.61 | 0.02 |
| C010R060 | MPC2 | Mitochondrial pyruvate carrier 2 | metabolic rate | -0.27 | 0.42 | 0.68 | 1.61 | 0.05 |
| C117R042 | MPC2 | Mitochondrial pyruvate carrier 2 | metabolic rate | -0.42 | 0.26 | 0.68 | 1.60 | 0.02 |
| C156R115 | MPC2 | Mitochondrial pyruvate carrier 2 | metabolic rate | -0.40 | 0.27 | 0.67 | 1.59 | 0.02 |
| C057R083 | PPT2 | lysosomal thioesterase PPT2-A-like | metabolic rate | 0.02 | 0.68 | 0.66 | 1.58 | 0.00 |
| C111R151 | HMGB3 | High mobility group protein B3 | immunity | 0.30 | 0.93 | 0.63 | 1.55 | 0.02 |
| C010R151 | CYCS | Cytochrome c, somatic | metabolic rate | -0.48 | 0.15 | 0.63 | 1.55 | 0.03 |
| C208R060 | CHCHD7 | Coiled-coil-helix-coiled-coil-helix domain-containing protein 7 | metabolic rate | 0.06 | 0.69 | 0.63 | 1.54 | 0.04 |
| C142R087 | ALAD | Delta-aminolevulinic acid dehydratase | oxygen transport | -0.60 | 0.02 | 0.62 | 1.54 | 0.03 |
| C066R146 | MRPS17 | 28S ribosomal protein S17 | growth | -0.31 | 0.31 | 0.62 | 1.54 | 0.02 |
| C167R048 | BCKDK | 3-methyl-2-oxobutanoate dehydrogenase [lipoamide] kinase | metabolic rate | 0.08 | 0.70 | 0.62 | 1.54 | 0.00 |
| C204R009 | UBALD1 | UBA-like domaing containing 1 | immunity | -0.46 | 0.16 | 0.62 | 1.54 | 0.00 |
| C175R095 | CCDC56 | Coiled-coil domain-containing protein 56 | metabolic rate | 0.04 | 0.64 | 0.61 | 1.52 | 0.02 |
| C172R080 | TOMM6 | Translocase of outer mitochondrial membrane 6 | immunity | -0.28 | 0.32 | 0.60 | 1.52 | 0.02 |
| C224R050 | PSMD9 | 26S proteasome non-ATPase regulatory subunit 9 | growth | -0.21 | 0.38 | 0.60 | 1.51 | 0.03 |
| C251R097 | TMEM254 | Transmembrane protein 254 | structural integrity | -0.14 | 0.45 | 0.59 | 1.51 | 0.04 |
| ***C216R021*** | ***NDUFB4*** | ***NADH dehydrogenase 1 beta subunit 4*** | metabolic rate | -0.22 | 0.37 | 0.59 | 1.51 | 0.03 |
| C211R097 | COX17 | Cytochrome c oxidase copper chaperone | metabolic rate | -0.22 | 0.36 | 0.58 | 1.50 | 0.03 |
| C238R060 | MRPL37 | 39S ribosomal protein L37 | growth | -0.18 | 0.35 | 0.53 | 1.44 | 0.04 |
| C045R135 | COX7A2L | Cytochrome c oxidase subunit VIIa-related protein | metabolic rate | -0.01 | 0.51 | 0.52 | 1.44 | 0.04 |
| C101R159 | TMED2 | Transmembrane emp24 domain-containing protein 2 precursor | structural integrity | -0.10 | 0.41 | 0.51 | 1.42 | 0.02 |
| C096R011 | ALAD | Delta-aminolevulinic acid dehydratase | oxygen transport | -0.55 | -0.07 | 0.48 | 1.40 | 0.01 |
| C245R002 | UQCRC2 | Cytochrome b-c1 complex subunit 2 | metabolic rate | 0.03 | 0.47 | 0.44 | 1.36 | 0.05 |
| C184R070 | ARPC5L | Actin-related protein 2/3 complex subunit 5-like protein | structural integrity | -0.03 | 0.39 | 0.41 | 1.33 | 0.02 |
|  |  |  |  |  |  |  |  |  |
| Downregulated in smolt | |  |  |  |  |  |  |  |
| ***C240R068*** | ***CCL4*** | ***C-C motif chemokine 4*** | immunity | 1.19 | -0.55 | -1.74 | -3.33 | 0.00 |
| ***C017R076*** | ***GlyT2*** | ***NA- and Cl-dependent glycine transporter 2*** | ion regulation | 0.84 | -0.80 | -1.64 | -3.13 | 0.02 |
| ***C188R011*** | ***CCL19*** | ***C-C motif chemokine 19*** | immunity | 0.92 | -0.59 | -1.51 | -2.86 | 0.00 |
| ***C260R153*** | ***IFI44*** | ***Interferon-induced protein 44*** | immunity | 0.40 | -0.78 | -1.18 | -2.27 | 0.00 |
| C124R109 | At5g5010 | Uncharacterized protein At5g50100 | growth | 0.41 | -0.67 | -1.09 | -2.12 | 0.02 |
| ***C023R137*** | ***MS4A4A*** | ***Membrane-spanning 4-domains A-4A*** | immunity | 0.40 | -0.58 | -0.99 | -1.98 | 0.02 |
| ***C164R090*** | ***PLK2*** | ***Serine/threonine-protein kinase PLK2*** | immunity | 0.31 | -0.64 | -0.95 | -1.93 | 0.01 |
| ***C241R010*** | ***CD3Z*** | ***T-cell surface glycoprotein CD3 zeta chain precursor*** | immunity | 0.19 | -0.74 | -0.93 | -1.90 | 0.01 |
| ***C230R063*** | ***UBA1*** | ***Ubiquitin-like modifier-activating enzyme 1 X*** | immunity | 0.49 | -0.43 | -0.92 | -1.89 | 0.04 |
| C244R027 |  | UNKNOWN |  | 0.29 | -0.62 | -0.91 | -1.88 | 0.02 |
| ***C090R027*** | ***EXO1*** | ***Exonuclease 1*** | immunity | 0.11 | -0.78 | -0.89 | -1.85 | 0.00 |
| C245R123 |  | UNKNOWN |  | 0.25 | -0.61 | -0.86 | -1.81 | 0.02 |
| ***C259R043*** | ***NAMPT*** | ***Nicotinamide phosphoribosyltransferase*** | immunity | 0.14 | -0.62 | -0.76 | -1.70 | 0.04 |
| ***C095R005*** | ***IL12B*** | ***Interleukin-12 beta*** | immunity | 0.31 | -0.43 | -0.74 | -1.67 | 0.00 |
| C223R096 | KDM5B | Lysine demethylase 5B | immunity | 0.34 | -0.36 | -0.70 | -1.62 | 0.04 |
| C164R017 | BHLHE40 | Basic helix-loop-helix family member E40 | repressor of circadian rhythm | -0.26 | -0.91 | -0.65 | -1.57 | 0.03 |
| C081R077 | NFX1 | Nuclear transcription factor, X-box binding 1 | immunity | 0.19 | -0.44 | -0.62 | -1.54 | 0.03 |
| C146R113 | DTX3 | Deltex E3 ubiquitin ligase 3 | immunity | 0.15 | -0.46 | -0.61 | -1.52 | 0.02 |
| C229R071 | ASAP2 | ArfGAP with SH3 domain, ankyrin repeat and PH domain 2 | immunity | 0.25 | -0.36 | -0.61 | -1.52 | 0.01 |
| C124R132 | RIPK4 | Receptor interacting serine-threonine kinase 4 | immunity | 0.31 | -0.27 | -0.58 | -1.50 | 0.01 |
| C057R087 |  | Transposable element |  | 0.05 | -0.50 | -0.54 | -1.46 | 0.03 |
| ***C124R129*** | ***MCM4*** | ***DNA replication licensing factor MCM4-B*** | immunity | -0.06 | -0.55 | -0.49 | -1.41 | 0.04 |
| C177R004 | BCL10 | B-cell lymphoma/leukemia 10 | immunity | 0.08 | -0.40 | -0.48 | -1.40 | 0.04 |

Table S2. Summary of the results from the 16K signature analysis using the Sockeye salmon and Rainbow trout datasets for gill tissue.

The Rainbow trout dataset was from Sutherland et al. (2014). Presented are the mapped 44K features that were significant for both Sockeye salmon and Rainbow trout, which are ordered by fold change. Bold italics are gene names with Probe ID used for qPCR assay development; normal italics are gene names and Probe IDs matching to same gene ID.

| Probe ID | Gene symbol | Gene name | Functional group | 16K study | Sockeye salmon | |  | Rainbow trout | |
| --- | --- | --- | --- | --- | --- | --- | --- | --- | --- |
|  |  |  |  |  | Log_2_ fold change | *P*-value |  | Log_2_ fold change | *P*-value |
| Upregulated in smolt | |  |  |  |  |  |  |  |  |
| ***C213R123*** | ***TSPO*** | ***Translocator protein*** | immunity | Robertson and McCormick (2012) | 1.04 | <0.01 |  | 0.50 | <0.01 |
| *C071R152* | *TSPO* | *Translocator protein* | immunity | Robertson and McCormick (2012) | 0.70 | <0.01 |  | 0.44 | <0.01 |
| *C030R160* | *TSPO* | *Translocator protein* | immunity | Robertson and McCormick (2012) | 0.64 | <0.01 |  | 0.54 | <0.01 |
| *C004R095* | *TSPO* | *Translocator protein* | immunity | Robertson and McCormick (2012) | 0.61 | <0.01 |  | 0.45 | <0.01 |
| C183R077 | SGK3 | serine/threonine kinase 3 | ion regulation | Robertson and McCormick (2012) | 0.48 | <0.01 |  | 0.66 | 0.04 |
| C057R039 | CYCS | Cytochrome C, somatic | metabolic rate | Boulet et al. (2012) | 0.48 | 0.04 |  | 0.98 | <0.01 |
| ***C037R160*** | ***NDUFB2*** | ***NADH dehydrogenase 1 beta subunit 2*** | metabolic rate | Robertson and McCormick (2012) | 0.29 | 0.02 |  | 0.26 | 0.02 |
|  |  |  |  |  |  |  |  |  |  |
| Downregulated in smolt | |  |  |  |  |  |  |  |  |
| ***C218R157*** | ***TUBA8L2*** | ***Tubulin, alpha 8 like 2*** | immunity | Lemmetyinen et al. (2013) | -0.73 | <0.01 |  | -0.28 | 0.04 |
| ***C217R022*** | ***FMNL1*** | ***Formin−like protein 1*** | immunity | Lemmetyinen et al. (2013) | -0.62 | <0.01 |  | -0.30 | 0.01 |
| C118R080 | PBRM1 | Polybromo-1 | immunity | Robertson and McCormick (2012) | -0.60 | 0.02 |  | -0.29 | 0.01 |
| *C217R123* | *TRA* | *T-cell receptor alpha* | immunity | Robertson and McCormick (2012) | -0.58 | 0.05 |  | -0.71 | 0.01 |
| ***C123R016*** | ***TRA*** | ***T-cell receptor alpha*** | immunity | Robertson and McCormick (2012) | -0.56 | 0.03 |  | -0.81 | <0.01 |
| *C189R144* | *TRA* | *T-cell receptor alpha* | immunity | Robertson and McCormick (2012) | -0.54 | 0.03 |  | -0.74 | 0.01 |
| *C185R169* | *TRA* | *T-cell receptor alpha* | immunity | Robertson and McCormick (2012) | -0.52 | <0.01 |  | -0.63 | 0.01 |
| *C062R132* | *FMNL1* | *Formin−like protein 1* | immunity | Lemmetyinen et al. (2013) | -0.51 | 0.03 |  | -0.39 | 0.03 |
| *C135R158* | *FMNL1* | *Formin−like protein 1* | immunity | Lemmetyinen et al. (2013) | -0.50 | 0.04 |  | -0.35 | 0.01 |
| ***C124R129*** | ***MCM4*** | ***DNA replication licensing factor MCM4−B*** | immunity | Robertson and McCormick (2012) | -0.49 | <0.01 |  | -0.57 | <0.01 |
| ***C058R025*** | ***TYK2*** | ***Non-receptor tyrosine-protein kinase TYK2*** | immunity | Robertson and McCormick (2012) | -0.46 | 0.01 |  | -0.34 | <0.01 |
| ***C052R071*** | ***WAS*** | ***Wiskott−Aldrich syndrome protein*** | immunity | Robertson and McCormick (2012) | -0.42 | 0.01 |  | -0.77 | <0.01 |
| *C236R165* | *TYK2* | *Non-receptor tyrosine-protein kinase TYK2* | immunity | Robertson and McCormick (2012) | -0.36 | 0.03 |  | -0.42 | <0.01 |
| *C131R157* | *TYK2* | *Non-receptor tyrosine-protein kinase TYK2* | immunity | Robertson and McCormick (2012) | -0.36 | 0.02 |  | -0.40 | <0.01 |

Table S3. Summary of the gene names associated with smoltification across published microarray studies using gill tissue.

Presented are generalized gene names organized by smoltification functional group for microarray studies including Atlantic salmon (*Salmo salar*), Brook trout (*Salvelinus fontinalis*), and Rainbow trout (*Oncorhynchus mykiss*). The symbol x indicates that a gene name was significant for separating parr and smolt.

|  | Seear et al. (2010) | Boulet et al. (2012) | Robertson and McCormick (2012) | Lemmetyinen et al. (2013) | Sutherland et al. (2014) |
| --- | --- | --- | --- | --- | --- |
| species | Atlantic salmon | Brook trout | Atlantic salmon | Atlantic salmon | Rainbow trout |
| platform | TRAITS/SGP | cGRASP 16K | cGRASP 16K | cGRASP 16K | cGRASP 44K |
|  |  |  |  |  |  |
| **Upregulated in smolt** |  |  |  |  |  |
|  |  |  |  |  |  |
| *Ion regulation* |  |  |  |  |  |
| Na^+^/K^+^-ATPase α-1b (seawater) | x |  | x | x | x |
| Cystic fibrosis transmembrane conductance regulator I |  |  | x |  | x |
|  |  |  |  |  |  |
| *Oxygen transport* |  |  |  |  |  |
| Hemoglobin (e.g. α and β) | x |  | x | x |  |
|  |  |  |  |  |  |
| *Metabolic rate (oxidative phosphorylation)* |  |  |  |  |  |
| NADH dehydrogenase | x | x | x |  | x |
| ATP synthase | x | x |  | x | x |
| Cytochrome c and cytochrome c oxidase | x |  | x | x | x |
| Glyceraldehyde-3-phosphate dehydrogenase |  | x |  |  | x |
|  |  |  |  |  |  |
| *Structural integrity* |  |  |  |  |  |
| Actin (e.g. β actin, cytoplasmic actin-1) |  |  | x | x |  |
| Collagen (e.g. α1I and α1X) | x |  |  | x | x |
| Myosin (e.g. tropomyosin α1) | x | x |  | x |  |
|  |  |  |  |  |  |
| *Body growth* |  |  |  |  |  |
| Elongation factor 2 | x |  |  | x |  |
| Glutamine synthetase |  |  | x |  | x |
| Ribosomal proteins (e.g. 40S, 60S) |  | x | x | x | x |
|  |  |  |  |  |  |
| *Immunity* |  |  |  |  |  |
| C-type lectin (e.g. 2 and 4m) | x | x | x | x | x |
| CC Chemokine (e.g. SCYA113) | x |  |  |  | x |
|  |  |  |  |  |  |
| **Downregulated in smolt** |  |  |  |  |  |
|  |  |  |  |  |  |
| *Immunity* |  |  |  |  |  |
| CC chemokine (e.g. SCYA112) | x |  |  | x | x |
| Regulator of G-protein (e.g. 1) |  |  |  | x | x |
| Keratin (e.g. type 12, 13) | x |  | x | x |  |
| Wiskott-Aldrich Syndrome protein |  |  | x |  | x |
| DNA replication licensing factor (e.g. MCM4-B) |  |  | x |  | x |
| Serine/threonine-protein phosphatase or kinase (e.g. PLK2) |  |  |  | x | x |

Table S4. Summary of qPCR TaqMan assay designs for candidate smoltification genes.

Presented are the forward, reverse, and TaqMan probe sequences, as well as the amplicon size. Assay names use the symbols described in Table 2 in main text. Two assays were designed for the top 12 upregulated and 10 downregulated genes (set 1), remaining genes had one assay design (set 2): v1 is version 1 and v2 is version 2.

| Assay name | Forward | | Reverse | | | Probe | Amplicon size (bp) |
| --- | --- | --- | --- | --- | --- | --- | --- |
| ***Upregulated in smolt*** | |  | | |  | |  |
| NKAa1-b_v1 | TGAAGAAGTGGTGGTTGGAGATC | | GGCAGAGACAATACGCAAATCA | | | TGAAAGGAGGAGATAGAAT | 79 |
| NKAa1-b_v2 | GCCTGGTGAAGAATCTTGAAGCT | | GAGTCAGGGTTCCGGTCTTG | | | CCTCCACCATTTGCTCA | 81 |
| CA4_v1 | GGTCATTTTGGTTTTGTACACAGTCT | | CCTAGATATAGCTATCCACGTACTCACCTA | | | TGATACGTGGTATAGAAAAG | 86 |
| CA4_v2 | CGTTACCTTGGCTCCCTGACT | | CTTGAACACGGTCCAAACCA | | | TCCAAATTGCAACGAGG | 63 |
| CFTR-I_v1 | GAGCTGTCAGAGAGGAAGTTCTCA | | GCAGCGACTCTTCAACCTGAT | | | TGGTGCCCGAGGAC | 61 |
| CFTR-I_v2 | ACGCCTGTCCAAAGATAGTGTCTA | | GCAAAGCATTGCTCCATATCC | | | AGCGAGGATGTGGACG | 72 |
| RHAG_v1 | GGTAGCTATGGCGATTGGATTT | | AATGTATCGGGATCCCAAGGT | | | TGCTGCACTGATATC | 61 |
| NKCC_v1 | GACACTACTGTGCTCTGTCCAAGAG | | TCAACATGGCTAGGCCACAA | | | CCAAAAGATGTTGCCTGTAA | 67 |
| GAPDH_v1 | GTGTCCACAGAACTTCACAGTGACT | | TGGTCGTTGAGTGCAATTCC | | | ATCTTTGACGCCGGTGC | 74 |
| GAPDH_v2 | CGGACATCCCACCAATCC | | TGTTGCCTTGGAGTTCACTACAG | | | CACCAGAGAAAAAGAG | 60 |
| NDUFB2_v1 | GCTTCTTACACGTGGACCTCAGA | | GCCTGTACTGGGCCTCTATGTG | | | CGAAAAGCTGGTGGTGGA | 77 |
| NDUFB2_v2 | TCATAGCGCTCGTTGTTGGTT | | TGTTTTCTCAAAATGTCGTTCCA | | | TCGCCCTCTGTGTCAA | 64 |
| NDUFB4_v1 | AAACAACCCGCACAGAAAAGA | | GGGTTGGTGCGTGCATACA | | | CTTATTGAAGACCCTGCCTT | 72 |
| MPC1_v1 | GCAGGCACTTTGGCACGTA | | TGAGATACTCTCTGAACTCCTTGCTT | | | AGCTGTTGACCATCTTA | 62 |
| HBA_v1 | GCCCTGGCTGACAAATACAGA | | GAGCAGGAACTGGAGTCCAATG | | | ACCATCATGAAAGTCC | 65 |
| HBA_v2 | GCTGCCCTGGCTGACAAA | | GGAACTGGAGTCCAATGATGGA | | | ACAGATAAGACCATCATGAAA | 63 |
| HBAt_v1 | TGGCTGACAAATACAGATAAGACCAT | | TTGTGATAACAACAGAGCAGAAACAG | | | ATGAAAGTCCAAACTTG | 75 |
| ACTB_v1 | GAAATCGCCGCACTGGTT | | CGGCGAATCCGGCTTT | | | TTGACAACGGATCCGGT | 58 |
| ACTB_v2 | GAAATCGCCGCACTGGTT | | GACGCCCCACGATGGA | | | CCGGAGATGACGCGC | 103 |
| WHRN_v1 | GGCTTTAGCAGGACTTTGCACTT | | CATCTCCCTCCTGTTCTCTCCTT | | | AAAGAGGTGAAACGGAAGG | 68 |
| RPL31_v1 | GAGTACACGGTCAACATCCACAA | | CGAGGTGCCCTCCTCTTAAA | | | CGCATACATGGCGTCT | 62 |
| RPL31_v2 | CGAAAGTTCGCCATGAAGGA | | CCTTGTTCAGGCGAGTATCGA | | | TGGGAACCCCTGACGTG | 64 |
| SLC16A10_v1 | ACTGCGGAGGAGCCTGAGTT | | ACATGGCAGCCAGCATAACC | | | CACCCAGAAGGAGGATG | 70 |
| EEF2_v1 | CCACCAAGATTTCCCAGATTGT | | TGCAGGAATTCCCTCCTTGA | | | TGACACACGTAAACGC | 68 |
| CYP2K1_v1 | ACCAGCCAAGACGTCACCTT | | CAGACGTCAGTAGAGGTAACACATCA | | | CAGGGATACTTCATCAAA | 73 |
| CYP2K1_v2 | GGAAGAACCTGCCCTACACTGA | | GGATGGACATGGGTACAATGTTG | | | TGATCCATGAGACCCAAA | 75 |
| S100A4_v1 | TGGCGCTGTGGACTTCAAG | | AGGAAGCAGTTGCACATGCA | | | AGTACATCACCCTGATTGA | 69 |
| TSPO_v1 | GGTGAAAACCTGGTACACCACTCT | | TGGGAACACAGCATTATTTGGA | | | AACAAGCCGTCATGGC | 65 |
| TSPO_v2 | CGATCTTCTTTGGAGCACACAA | | AACAGCACCAGTCAACATCACTATC | | | TTGAAAATGGCACTCATAG | 68 |
| CLEC4M_v1 | CGTGGCACTGAACTCTTATGCA | | GGTACAGCCACTGTCACACTTGA | | | TGAGGGCTTCGATGAC | 67 |
| CLEC4M_v2 | GGGCTTCGATGACATCAAGTG | | ACTCCACCAGTAGCAATTCTCATG | | | CAGTGGCTGTACCTTT | 69 |
| RGS5_v1 | CAGCATTTCTGTTTGTCACTAACCA | | CCCTCAAAACCATCTACTTCTCTGA | | | TCACTATAATAACTAGCCTTCACAC | 85 |
| FKBP5_v1 | GGGCGTTCCTCTGGGTGTA | | GCATGCAGCATTCTCCTTTCT | | | ACAGGGCCATGGAGA | 62 |
| THRB1_v1 | GGCTAAGTCAATGGCCCTAGAA | | TGTCTCAAACGAATTGCCTCAA | | | AGTTGTGACAATCAGC | 66 |
| THRB1_v2 | TGCATGGAGATCATGTCTTTACG | | CGGCCATCTCCCCGTTA | | | AGAGTGAGACGCTGACG | 82 |
| GHR1_v1 | CCGTTCCTTCTCCCAACATG | | TGTACTCACCATACATCTTACCAAACC | | | CAGCTGTAGACATTTT | 66 |
| NR3C1_v1 | TTGGACTTGCCTGGCTCTCT | | GACAGGGAGGAAAGGAAAGCA | | | ACCTGAATGAGTTTTACGTGTC | 77 |
|  |  | |  | | |  |  |
| ***Downregulated in smolt*** | |  | |  | | |  |
| NKAa1-a_v1 | GAACAGAACAACGTACCTATCCTCAA | | TTGAGGATAGGTACGTTGTTCTGTTC | | | CTGTGCAACCGAGCC | 79 |
| NKAa1-a_v2 | TGGAATCAAGGTTATCATGGTCACT | | CCCACACCCTTGGCAATG | | | ATCATCCCATCACTGCGA | 69 |
| GlyT2_v1 | TTCCTTGTCGTTTTCCTGTTCTC | | CACCCACATTGAATGGAAAGAG | | | ACTTCATCTCTCCTTTCC | 72 |
| GlyT2_v2 | CACTTCACTTCATCTCTCCTTTCCT | | GGATGTGTTGGCTTGCTTTTC | | | TTTCCATTCAATGTGGGTGC | 73 |
| CCL4_v1 | TCTCTTCATTGCAACAATCTGCTT | | ACAGCAGTCCACGGGTACCT | | | CTACGCAGCAGCATT | 67 |
| CCL4_v2 | GACTGCTGTCTGTCAACCACTGA | | CTGCAGCAGGTAGGAGACCAT | | | TTTCCCTCGCCACTTT | 70 |
| CCL19_v1 | ACCTGGGTTACAGACCTGATGAA | | TGGTTTCGTGGCATTTCTTG | | | CTCATGGACCGCCTCA | 61 |
| IFI44_v1 | AGGAAACTGCCCGTGACATC | | GGTCATCGTCTGCTGAACGA | | | CCCAGGTGTACAACAGT | 62 |
| IFI44_v2 | CCACTGGACTAACCCTCCATGA | | TGTGTCCCTCGGGTGCAT | | | ACTCTGGCTATCATCAAA | 63 |
| PLK2_v1 | AACAATGGCACACACATGAGTCT | | CCCAGCTCGGCATAGTAGTGA | | | CTGGCAGATAAGAGGAC | 65 |
| PLK2_v2 | CTGGGCAGCAGCTTCCA | | TGGTAGCCAAAGCCATACTTGTT | | | TGACCAAATGGGTGGACT | 68 |
| CD3Z_v1 | CCCTGCAGAGGCGATGAC | | GGAGGAAGCCATCCAGAATGT | | | CCACTGTACGACCCTAA | 65 |
| CD3Z_v2 | CCCTGCAGAGGCGATGAC | | GGAGGAAGCCATCCAGAATGT | | | CGACCCTAAACTCTG | 65 |
| IL12B_v1 | GGAGCCTCCCATGCTCTTACT | | TGGCGTGGACCACTTTGAC | | | CCCCTCACATTCCA | 57 |
| IL12B_v2 | GGGAACCAGATCCAGGTGAA | | AGCGTTGTGGCAGCTGTAGTT | | | TGGAGGAGATGATGGGA | 63 |
| MCM4_v1 | AAGCTGGAGGAGATCAGTGTGATT | | TGCACATGGCTGCAGTTGA | | | TGAGCCTGTGCTGAAT | 62 |
| MCM4_v2 | GCTGCCAAGTGCAGATGGT | | GACATCAGTGCCCCAGATAACC | | | CAGGCAGCGGGACA | 76 |
| WAS_v1 | GCAGGAGCTCTATAACCAAATGGT | | CAGCAAATGCGTGGAAGAAG | | | TACCACAGCCCCCGAC | 62 |
| WAS_v2 | GCATTCCATCCTGTATTATTTTCCA | | GAGATTAACCGGATCAACCAGACT | | | TCTGTAACCATGGACTCAA | 70 |
| TYK2_v1 | GGGAGTGGCCAAAAGTGTCTT | | CGTGAAGCGCATAGATAGAGAAGTC | | | CGGTGTTTTCGTCTTGTAG | 68 |
| TYK2_v2 | CGCATGTGATAAATACGACTTGATT | | TCCCCTTGAACCAGCTGTACTAC | | | AGCTATACCACAAGCGTAC | 70 |
| RGS21_v1 | TCCCGACTACAGCGCAGAT | | TCCTCAGGGCTAAGTCGTTCA | | | TTCCCAATCCCCC | 59 |
| MS4A4A_v1 | TGGGCAGTGTTGAAGATTCTGA | | GCATGGTTCTGCCTCAAGGA | | | CCCCGGCTCCACCA | 63 |
| UBA1_v1 | TTGAGGGCAGAGAACTACGACAT | | TGCGACCCGCGATCA | | | CTGATCGTCACAAGAGT | 67 |
| EXO1_v1 | GTGGTGGCCCCGTATGAA | | CAATGCCAGCCTTGTTAAGGA | | | CTGATGCCCAGCTGG | 58 |
| NAMPT_v1 | CCAACACACTACGCAGAAAGGA | | CCTTGGTGGTCAGTGCTGTACA | | | TGGATCCCTTCTACCTCC | 64 |
| TUBA8L2_v1 | CCCGCGCCATCTTTGTAG | | AACAGCTGGCGGTAGGTACCT | | | ACTGTCATTGATGAGGTGC | 73 |
| FMNL1_v1 | GTTTGGATGTACTGGTGGATTACCT | | GCACCCTCCAAGTCAAACGA | | | CTACGCCCAGTGTGAC | 67 |
| TRA_v1 | TTCAGCGCACACAATGCTACT | | TGGTGGCCTCTGTCTTGTTG | | | CGCACTTGGAATCC | 58 |
| PRLR_v1 | GATGCCGGAGGGAAAAGAC | | CCGACTGGCTCTTGGACTTG | | | TCCAAGATGTTGGCTGC | 59 |

Table S5. Summary of efficiency values for the qPCR TaqMan assay designs using six to nine salmonid species.

Species abbreviations: CK= Chinook salmon (*Oncorhynchus tshawytscha*), CO= Coho salmon (*O. kisutch*), SX= Sockeye salmon (*O. nerka*), PK= Pink salmon *(O.* *gorbuscha*), CM= Chum salmon (*O. keta*), AS= Atlantic salmon (*Salmo salar*), RT= Rainbow trout (*O. mykiss*), AC= Arctic charr (*Salvelinus alpinus*), and BT= Bull trout (*Salvelinus confluentus*). Both assay designs of set 1 were tested for efficiency using six species. The best assay design of set 1 and all of set 2 single assay designs were then tested using nine species. Efficiency values between 0.8 and 1.2 were considered good. FAIL indicates no detectable amplification. See Table 2 legend in main text for additional details.

| Assay name | Selected | CK | CO | SX | PK | CM | AS | RT | AC | BT |
| --- | --- | --- | --- | --- | --- | --- | --- | --- | --- | --- |
| ***Upregulated in smolt*** | |  |  |  |  |  |  |  |  |  |
| NKAa1-b_v1 |  | 0.870 | 1.010 | 0.844 | 1.304 | 0.865 | 0.923 |  |  |  |
| NKAa1-b_v2 | yes | 1.156 | 1.044 | 1.073 | 1.051 | 1.122 | 1.068 | 1.086 | 0.987 | 1.067 |
| CA4_v1 | yes | 0.999 | 1.055 | 1.011 | 0.981 | 1.090 | 1.149 | 0.937 | FAIL | 0.918 |
| CA4_v2 |  | 0.872 | 1.138 | 0.855 | 0.945 | 0.911 | FAIL |  |  |  |
| CFTR-I_v1 | yes | 0.980 | 0.704 | 1.046 | 0.999 | 1.126 | 0.969 | 1.183 | 0.882 | 1.078 |
| CFTR-I_v2 |  | 0.946 | 1.512 | 0.908 | 0.537 | 1.770 | 3.891 |  |  |  |
| RHAG_v1 | yes | 1.046 | 1.097 | 1.099 | 1.003 | 1.125 | 0.953 | 1.019 | 0.967 | 0.999 |
| NKCC_v1 |  | FAIL | FAIL | FAIL | FAIL | FAIL | FAIL | 0.887 | FAIL | FAIL |
| GAPDH_v1 |  | 1.069 | FAIL | 0.898 | FAIL | 0.896 | FAIL |  |  |  |
| GAPDH_v2 |  | FAIL | FAIL | FAIL | FAIL | FAIL | FAIL |  |  |  |
| NDUFB2_v1 | yes | 0.992 | 0.972 | 1.029 | 1.023 | 1.060 | 1.100 | 1.042 | 0.921 | 1.009 |
| NDUFB2_v2 |  | 1.164 | 0.934 | 0.774 | 0.757 | FAIL | 1.352 |  |  |  |
| NDUFB4_v1 | yes | 1.029 | 1.129 | 1.137 | 1.019 | 1.218 | 0.953 | 1.090 | 0.991 | 1.143 |
| MPC1_v1 | yes | 1.005 | 1.044 | 1.187 | 1.163 | 1.166 | 1.127 | 1.039 | 1.108 | 1.029 |
| HBA_v1 | yes | 1.200 | 0.995 | 1.070 | 1.248 | 1.111 | 1.630 | 1.048 | FAIL | FAIL |
| HBA_v2 |  | 0.851 | 0.849 | 0.862 | 1.099 | 0.802 | 1.042 |  |  |  |
| HBAt_v1 | yes | 1.081 | 0.946 | 0.997 | 1.142 | 1.187 | FAIL | 1.008 | FAIL | FAIL |
| ACTB_v1 | yes | 1.210 | 1.050 | 1.071 | 1.046 | 1.192 | 1.167 | 1.260 | 1.251 | 1.070 |
| ACTB_v2 |  | 0.806 | 1.100 | 0.979 | 1.406 | 1.177 | 1.542 |  |  |  |
| WHRN_v1 |  | FAIL | 1.385 | 0.825 | 3.648 | 4.090 | 1.025 | FAIL | 1.187 | 2.553 |
| RPL31_v1 | yes | 0.993 | 0.974 | 1.003 | 0.983 | 1.009 | 1.342 | 1.072 | 0.913 | 1.000 |
| RPL31_v2 |  | 0.858 | 0.863 | 0.845 | 1.257 | 0.857 | 1.372 |  |  |  |
| SLC16A10_v1 | yes | 0.901 | 0.963 | 1.103 | 0.885 | 1.057 | 0.893 | 1.283 | 0.895 | 0.915 |
| EEF2_v1 | yes | 0.940 | 1.037 | 1.020 | 0.972 | 1.089 | 1.244 | 1.138 | 1.024 | 1.096 |
| CYP2K1_v1 |  | 0.834 | 0.855 | 0.872 | 0.702 | 1.275 | 1.409 |  |  |  |
| CYP2K1_v2 | yes | 0.937 | 0.933 | 1.029 | 0.966 | 0.811 | 1.130 | 0.828 | 0.810 | 1.525 |
| S100A4_v1 |  | FAIL | 1.128 | FAIL | 1.491 | 1.428 | 1.755 | 1.691 | 1.432 | 1.188 |
| TSPO_v1 |  | 0.835 | 0.850 | 0.844 | 1.139 | 0.829 | FAIL |  |  |  |
| TSPO_v2 | yes | 0.920 | 0.845 | 0.880 | 0.936 | 0.980 | 0.890 | 0.966 | 0.875 | 0.853 |
| CLEC4M_v1 | yes | 1.030 | 0.875 | 0.975 | 1.106 | 1.153 | 1.031 | 1.137 | 1.519 | FAIL |
| CLEC4M_v2 |  | 0.955 | 1.063 | 0.685 | 0.819 | 0.951 | 0.819 |  |  |  |
| RGS5_v1 |  | 1.298 | 0.982 | 1.176 | 1.228 | 1.272 | 1.358 | 1.220 | 1.025 | 1.151 |
| FKBP5_v1 | yes | 0.867 | 0.963 | 1.019 | 0.713 | 0.964 | 0.977 | 0.897 | 0.847 | 0.880 |
| THRB1_v1 |  | 0.796 | 0.806 | 0.792 | 1.054 | 0.784 | 0.760 |  |  |  |
| THRB1_v2 | yes | 0.841 | 0.724 | 0.848 | 0.964 | 0.997 | FAIL | 1.094 | FAIL | FAIL |
| GHR1_v1 | yes | 0.937 | 1.160 | 0.958 | 1.081 | 0.945 | 0.967 | 0.978 | 0.845 | 0.935 |
| NR3C1_v1 | yes | 1.082 | 0.993 | 1.046 | 0.937 | 1.028 | 1.106 | FAIL | 0.891 | 0.929 |
|  |  |  |  |  |  |  |  |  |  |  |
| ***Downregulated in smolt*** | |  |  |  |  |  |  |  |  |  |
| NKAa1-a_v1 |  | FAIL | FAIL | FAIL | FAIL | FAIL | FAIL |  |  |  |
| NKAa1-a_v2 | yes | 1.160 | 1.042 | 1.064 | 1.013 | 1.162 | 1.151 | 1.153 | 0.988 | 1.033 |
| GlyT2_v1 |  | FAIL | FAIL | FAIL | FAIL | FAIL | FAIL |  |  |  |
| GlyT2_v2 |  | FAIL | FAIL | FAIL | FAIL | FAIL | FAIL |  |  |  |
| CCL4_v1 | yes | 0.981 | 1.130 | 1.021 | 1.018 | 0.998 | 0.948 | 1.085 | 0.916 | 0.687 |
| CCL4_v2 |  | 0.879 | 1.027 | 0.856 | 1.314 | 1.159 | 0.839 |  |  |  |
| CCL19_v1 | yes | 1.010 | 0.954 | 1.019 | 0.973 | 1.084 | 1.110 | 1.003 | 0.970 | 1.026 |
| IFI44_v1 | yes | 1.096 | 1.028 | 1.012 | 1.079 | 1.089 | 1.185 | 1.083 | 1.060 | 1.131 |
| IFI44_v2 |  | 0.855 | 0.823 | 0.889 | 0.881 | 1.068 | 0.838 |  |  |  |
| PLK2_v1 |  | 0.902 | 0.957 | 1.457 | 0.865 | 0.895 | 0.966 |  |  |  |
| PLK2_v2 | yes | 0.931 | 0.866 | 1.578 | 0.847 | 0.972 | 1.063 | 0.983 | 1.370 | FAIL |
| CD3Z_v1 |  | 2.972 | 1.561 | 1.161 | 2.040 | 2.750 | 1.316 | FAIL | 1.137 | 0.776 |
| CD3Z_v2 |  | 1.321 | 1.054 | 2.097 | 1.256 | 1.147 | 1.308 |  |  |  |
| IL12B_v1 | yes | 0.967 | 0.931 | 0.846 | 0.881 | 0.805 | 0.885 | 0.976 | FAIL | FAIL |
| IL12B_v2 |  | 0.774 | FAIL | 0.766 | 1.228 | 0.916 | FAIL |  |  |  |
| MCM4_v1 | yes | 0.903 | 0.942 | 1.013 | 0.862 | 1.139 | 0.913 | 0.934 | 0.848 | 0.924 |
| MCM4_v2 |  | 0.855 | 0.828 | 0.933 | FAIL | 0.776 | 0.701 |  |  |  |
| WAS_v1 | yes | 1.012 | 1.028 | 0.969 | 0.955 | 0.999 | 1.251 | 1.049 | 0.870 | 1.003 |
| WAS_v2 |  | 0.774 | 0.716 | 0.803 | 0.816 | 0.872 | 2.125 |  |  |  |
| TYK2_v1 |  | FAIL | FAIL | FAIL | FAIL | FAIL | FAIL |  |  |  |
| TYK2_v2 |  | FAIL | FAIL | FAIL | FAIL | FAIL | FAIL |  |  |  |
| RGS21_v1 | yes | 0.867 | 0.838 | 1.099 | FAIL | FAIL | 0.993 | FAIL | 0.872 | 0.991 |
| MS4A4A_v1 | yes | 0.960 | 1.066 | 1.265 | 1.016 | 1.084 | 1.027 | 1.041 | 1.236 | 1.051 |
| UBA1_v1 | yes | 0.937 | 0.854 | 1.049 | 0.984 | 1.064 | 1.080 | 0.983 | 0.896 | 0.885 |
| EXO1_v1 | yes | 0.930 | 0.902 | 1.017 | 1.032 | 1.033 | 0.742 | 1.005 | 0.916 | 1.191 |
| NAMPT_v1 | yes | 0.978 | 1.029 | 1.170 | 1.066 | 1.020 | 1.105 | 1.024 | 1.193 | 0.965 |
| TUBA8L2_v1 | yes | 1.078 | 1.084 | 1.126 | 1.159 | 1.138 | 1.279 | 1.140 | 1.077 | 1.131 |
| FMNL1_v1 | yes | 1.059 | 0.867 | 0.967 | 1.176 | 1.225 | 1.208 | 1.127 | 1.043 | 1.058 |
| TRA_v1 | yes | 0.963 | 1.500 | 0.972 | 0.940 | 0.978 | FAIL | FAIL | FAIL | FAIL |
| PRLR_v1 | yes | 0.949 | 1.100 | 0.865 | 1.010 | 0.979 | 1.728 | 0.722 | 0.916 | FAIL |
