# Supplementary material for: Discovery and validation of candidate smoltification gene expression biomarkers across multiple species and ecotypes of Pacific salmonids
Source: Conserv Physiol. 2019 Oct 11;7(1):coz051. doi: 10.1093/conphys/coz051 (PMC6788492; doi:10.1093/conphys/coz051)
Supplement: suppl_analysis_coz051 [file suppl_analysis_coz051.docx]

Supplemental Analysis for **‘**Discovery and validation of candidate smoltification gene expression biomarkers across multiple species and ecotypes of Pacific salmonids’

Table S6. Summary of the Pearson correlations between PC2 and the expression of 37 candidate smoltification genes using all four groups.

Table S7. Summary of the Pearson correlations between PC2 and the expression of 37 candidate smoltification genes for Coho salmon.

Table S8. Summary of the Pearson correlations between PC2 and the expression of 37 candidate smoltification genes for Sockeye salmon.

Table S9. Summary of the Pearson correlations between PC2 and the expression of 37 candidate smoltification genes for stream-type Chinook salmon.

Table S10. Summary of the Pearson correlations between PC2 or PC3 and the expression of 37 candidate smoltification genes for ocean-type Chinook salmon.

Table S11. Summary of the Student’s t-tests examining the difference in expression of 37 candidate smoltification genes between freshwater and seawater for ocean-type Chinook salmon.

Table S12. Summary of body variable correlations with smoltification gene expression patterns for the four groups.

Table S6. Summary of the Pearson correlations between PC2 and the expression of 37 candidate smoltification genes using all four groups.

| Assay name | *r* | *p* |
| --- | --- | --- |
| ACTB_v1 | -0.03628 | 0.402779 |
| CA4_v1 | 0.650688 | 0 |
| CCL19_v1 | -0.45388 | 0 |
| CCL4_v1 | -0.37056 | 0 |
| CFTR.I_v1 | 0.723941 | 0 |
| CLEC4M_v1 | 0.343777 | 4.44E-16 |
| CYP2K1_v2 | 0.226706 | 1.19E-07 |
| EEF2_v1 | 0.414287 | 0 |
| EXO1_v1 | -0.16926 | 8.48E-05 |
| FKBP5_v1 | 0.518222 | 0 |
| FMNL1_v1 | -0.3929 | 0 |
| GHR1_v1 | 0.199101 | 3.54E-06 |
| HBA_v1 | 0.624489 | 0 |
| HBAt_v1 | -0.13112 | 0.002398 |
| IFI44_v1 | -0.56695 | 0 |
| IL12B_v1 | -0.32228 | 2.26E-14 |
| MCM4_v1 | -0.00672 | 0.876895 |
| MPC1_v1 | 0.147246 | 0.000642 |
| MS4A4A_v1 | -0.23536 | 3.72E-08 |
| NAMPT_v1 | -0.16426 | 0.000137 |
| NDUFB2_v1 | 0.302671 | 8.92E-13 |
| NDUFB4_v1 | 0.232095 | 5.79E-08 |
| NKAa1.a_v2 | -0.09342 | 0.030888 |
| NKAa1.b_v2 | 0.339919 | 6.66E-16 |
| NR3C1_v1 | -0.14933 | 0.000536 |
| PLK2_v2 | -0.15146 | 0.000445 |
| PRLR_v1 | -0.17944 | 3.04E-05 |
| RGS21_v1 | -0.10182 | 0.018594 |
| RHAG_v1 | 0.244954 | 9.76E-09 |
| RPL31_v1 | 0.06362 | 0.14205 |
| SLC16A10_v1 | 0.103012 | 0.017256 |
| THRB1_v2 | 0.068314 | 0.114846 |
| TRA_v1 | -0.22363 | 1.77E-07 |
| TSPO_v2 | 0.133854 | 0.001936 |
| TUBA8L2_v1 | -0.04732 | 0.275042 |
| UBA1_v1 | -0.15486 | 0.000328 |
| WAS_v1 | -0.4355 | 0 |

Table S7. Summary of the Pearson correlations between PC2 and the expression of 37 candidate smoltification genes for Coho salmon.

| Assay name | *r* | *p* |
| --- | --- | --- |
| ACTB_v1 | -0.1777 | 0.026462 |
| CA4_v1 | -0.86386 | 0 |
| CCL19_v1 | 0.243586 | 0.002182 |
| CCL4_v1 | 0.178953 | 0.025403 |
| CFTR.I_v1 | -0.88924 | 0 |
| CLEC4M_v1 | -0.0952 | 0.23714 |
| CYP2K1_v2 | -0.22418 | 0.004903 |
| EEF2_v1 | -0.50957 | 1.09E-11 |
| EXO1_v1 | 0.118789 | 0.139682 |
| FKBP5_v1 | -0.58557 | 8.88E-16 |
| FMNL1_v1 | 0.399286 | 2.43E-07 |
| GHR1_v1 | -0.1223 | 0.128273 |
| HBA_v1 | -0.7775 | 0 |
| HBAt_v1 | -0.66312 | 0 |
| IFI44_v1 | 0.377681 | 1.17E-06 |
| IL12B_v1 | 0.232621 | 0.003475 |
| MCM4_v1 | 0.435372 | 1.35E-08 |
| MPC1_v1 | -0.03564 | 0.658675 |
| MS4A4A_v1 | 0.107597 | 0.181235 |
| NAMPT_v1 | 0.266893 | 0.000757 |
| NDUFB2_v1 | -0.42869 | 2.36E-08 |
| NDUFB4_v1 | -0.38738 | 5.86E-07 |
| NKAa1.a_v2 | -0.25199 | 0.001507 |
| NKAa1.b_v2 | -0.68527 | 0 |
| NR3C1_v1 | 0.053141 | 0.509986 |
| PLK2_v2 | 0.000665 | 0.993427 |
| PRLR_v1 | -0.22419 | 0.0049 |
| RGS21_v1 | -0.23058 | 0.003781 |
| RHAG_v1 | -0.73892 | 0 |
| RPL31_v1 | -0.40186 | 2.00E-07 |
| SLC16A10_v1 | -0.39925 | 2.43E-07 |
| THRB1_v2 | -0.03892 | 0.629536 |
| TRA_v1 | 0.107788 | 0.180456 |
| TSPO_v2 | -0.05689 | 0.480554 |
| TUBA8L2_v1 | 0.054845 | 0.49649 |
| UBA1_v1 | 0.351538 | 6.80E-06 |
| WAS_v1 | 0.378703 | 1.09E-06 |

Table S8. Summary of the Pearson correlations between PC2 and the expression of 37 candidate smoltification genes for Sockeye salmon.

| Assay name | *r* | *p* |
| --- | --- | --- |
| ACTB_v1 | 0.082718 | 0.468612 |
| CA4_v1 | 0.793261 | 0 |
| CCL19_v1 | -0.22605 | 0.045165 |
| CCL4_v1 | -0.35073 | 0.00153 |
| CFTR.I_v1 | 0.832734 | 0 |
| CLEC4M_v1 | -0.38243 | 0.000505 |
| CYP2K1_v2 | 0.327642 | 0.003203 |
| EEF2_v1 | -0.51509 | 1.19E-06 |
| EXO1_v1 | -0.02941 | 0.796958 |
| FKBP5_v1 | 0.232226 | 0.039454 |
| FMNL1_v1 | -0.24315 | 0.030835 |
| GHR1_v1 | 0.490326 | 4.50E-06 |
| HBA_v1 | 0.813886 | 0 |
| HBAt_v1 | 0.682975 | 4.10E-12 |
| IFI44_v1 | -0.46057 | 1.95E-05 |
| IL12B_v1 | 0.592312 | 8.93E-09 |
| MCM4_v1 | -0.16452 | 0.147384 |
| MPC1_v1 | 0.454375 | 2.60E-05 |
| MS4A4A_v1 | 0.154063 | 0.175218 |
| NAMPT_v1 | 0.244134 | 0.03014 |
| NDUFB2_v1 | 0.621709 | 9.67E-10 |
| NDUFB4_v1 | 0.515412 | 1.17E-06 |
| NKAa1.a_v2 | 0.211878 | 0.060856 |
| NKAa1.b_v2 | 0.75616 | 8.88E-16 |
| NR3C1_v1 | 0.203574 | 0.071946 |
| PLK2_v2 | 0.09461 | 0.406893 |
| PRLR_v1 | 0.597026 | 6.35E-09 |
| RGS21_v1 | -0.41606 | 0.000137 |
| RHAG_v1 | 0.80588 | 0 |
| RPL31_v1 | -0.25337 | 0.024257 |
| SLC16A10_v1 | 0.398443 | 0.000276 |
| THRB1_v2 | 0.245944 | 0.028901 |
| TRA_v1 | -0.44742 | 3.56E-05 |
| TSPO_v2 | 0.733132 | 1.55E-14 |
| TUBA8L2_v1 | 0.265784 | 0.017912 |
| UBA1_v1 | -0.0791 | 0.488367 |
| WAS_v1 | -0.11068 | 0.331505 |

Table S9. Summary of the Pearson correlations between PC2 and the expression of 37 candidate smoltification genes for stream-type Chinook salmon.

| Assay name | *r* | *p* |
| --- | --- | --- |
| ACTB_v1 | 0.063431 | 0.47514 |
| CA4_v1 | -0.58541 | 3.19E-13 |
| CCL19_v1 | 0.709524 | 0 |
| CCL4_v1 | 0.621435 | 4.00E-15 |
| CFTR.I_v1 | -0.24342 | 0.00544 |
| CLEC4M_v1 | -0.33614 | 9.84E-05 |
| CYP2K1_v2 | 0.157624 | 0.074423 |
| EEF2_v1 | -0.31222 | 0.000316 |
| EXO1_v1 | 0.321477 | 0.000203 |
| FKBP5_v1 | -0.60248 | 4.24E-14 |
| FMNL1_v1 | 0.275064 | 0.001606 |
| GHR1_v1 | -0.22987 | 0.008777 |
| HBA_v1 | -0.56708 | 2.45E-12 |
| HBAt_v1 | -0.44971 | 8.97E-08 |
| IFI44_v1 | 0.746626 | 0 |
| IL12B_v1 | 0.385703 | 6.36E-06 |
| MCM4_v1 | 0.308279 | 0.000379 |
| MPC1_v1 | -0.16822 | 0.056697 |
| MS4A4A_v1 | 0.196692 | 0.025475 |
| NAMPT_v1 | 0.132866 | 0.133346 |
| NDUFB2_v1 | -0.13715 | 0.121179 |
| NDUFB4_v1 | -0.15982 | 0.070426 |
| NKAa1.a_v2 | -0.09251 | 0.297078 |
| NKAa1.b_v2 | -0.1793 | 0.042038 |
| NR3C1_v1 | 0.071568 | 0.420253 |
| PLK2_v2 | 0.101785 | 0.251057 |
| PRLR_v1 | -0.01846 | 0.835538 |
| RGS21_v1 | 0.224464 | 0.010548 |
| RHAG_v1 | -0.12244 | 0.166865 |
| RPL31_v1 | 0.051096 | 0.56525 |
| SLC16A10_v1 | 0.033999 | 0.702085 |
| THRB1_v2 | -0.2421 | 0.005707 |
| TRA_v1 | 0.462402 | 3.45E-08 |
| TSPO_v2 | -0.01029 | 0.90786 |
| TUBA8L2_v1 | 0.157099 | 0.075406 |
| UBA1_v1 | 0.10494 | 0.236586 |
| WAS_v1 | 0.308611 | 0.000373 |

Table S10. Summary of the Pearson correlations between PC2 or PC3 and the expression of 37 candidate smoltification genes for ocean-type Chinook salmon.

| Assay name | *r* (PC2) | *p* (PC2) | *r* (PC3) | *p* (PC3) |
| --- | --- | --- | --- | --- |
| ACTB_v1 | 0.295468 | 9.16E-05 | 0.111455 | 0.147902 |
| CA4_v1 | -0.82921 | 0 | -0.24564 | 0.001244 |
| CCL19_v1 | -0.45581 | 4.21E-10 | 0.368436 | 7.68E-07 |
| CCL4_v1 | -0.62148 | 0 | 0.380255 | 3.14E-07 |
| CFTR.I_v1 | -0.53029 | 1.03E-13 | -0.3243 | 1.60E-05 |
| CLEC4M_v1 | 0.295834 | 8.97E-05 | -0.3938 | 1.08E-07 |
| CYP2K1_v2 | 0.380222 | 3.15E-07 | -0.33119 | 1.03E-05 |
| EEF2_v1 | 0.131025 | 0.08855 | 0.158466 | 0.039022 |
| EXO1_v1 | 0.24312 | 0.0014 | -0.24796 | 0.001113 |
| FKBP5_v1 | -0.13548 | 0.078149 | 0.53799 | 3.86E-14 |
| FMNL1_v1 | 0.02361 | 0.759903 | -0.05587 | 0.469299 |
| GHR1_v1 | 0.567149 | 8.88E-16 | 0.106027 | 0.168788 |
| HBA_v1 | -0.23681 | 0.001876 | -0.30541 | 5.13E-05 |
| HBAt_v1 | -0.52606 | 1.75E-13 | 0.144307 | 0.060452 |
| IFI44_v1 | -0.20517 | 0.007275 | 0.51451 | 7.12E-13 |
| IL12B_v1 | -0.57207 | 4.44E-16 | -0.05122 | 0.507131 |
| MCM4_v1 | 0.264691 | 0.000486 | -0.31182 | 3.48E-05 |
| MPC1_v1 | 0.347345 | 3.47E-06 | 0.211596 | 0.005607 |
| MS4A4A_v1 | -0.04324 | 0.575596 | -0.11498 | 0.135411 |
| NAMPT_v1 | 0.057584 | 0.455739 | 0.419977 | 1.19E-08 |
| NDUFB2_v1 | 0.212028 | 0.005508 | 0.142497 | 0.063782 |
| NDUFB4_v1 | 0.281835 | 0.000197 | 0.189455 | 0.013345 |
| NKAa1.a_v2 | -0.0336 | 0.663534 | 0.27528 | 0.00028 |
| NKAa1.b_v2 | -0.26095 | 0.000588 | -0.1643 | 0.032274 |
| NR3C1_v1 | 0.200761 | 0.008663 | 0.094512 | 0.22022 |
| PLK2_v2 | -0.36256 | 1.18E-06 | -0.17241 | 0.024564 |
| PRLR_v1 | -0.25758 | 0.000696 | -0.02923 | 0.70518 |
| RGS21_v1 | -0.36651 | 8.85E-07 | -0.39598 | 9.04E-08 |
| RHAG_v1 | 0.206415 | 0.006921 | -0.14098 | 0.066685 |
| RPL31_v1 | 0.1849 | 0.015784 | -0.10159 | 0.187427 |
| SLC16A10_v1 | 0.251176 | 0.000953 | -0.16253 | 0.034209 |
| THRB1_v2 | -0.20179 | 0.008319 | 0.340011 | 5.72E-06 |
| TRA_v1 | -0.12969 | 0.09187 | -0.34609 | 3.78E-06 |
| TSPO_v2 | -0.22918 | 0.002645 | -0.3487 | 3.16E-06 |
| TUBA8L2_v1 | 0.375566 | 4.50E-07 | 0.279671 | 0.000221 |
| UBA1_v1 | -0.1985 | 0.009462 | 0.396463 | 8.69E-08 |
| WAS_v1 | -0.32037 | 2.05E-05 | -0.01946 | 0.801146 |

Table S11. Summary of the Student’s t-tests examining the difference in expression of 37 candidate smoltification genes between freshwater and seawater for ocean-type Chinook salmon.

Nitinat juveniles were sampled at the same time in late April in both environments. Seawater juveniles were contained within a netpen in an estuary for about two weeks prior to sampling. Presented are the mean values in freshwater and seawater; *t* and *p*-values for the mean difference; and significance coding for the difference, i.e. *** *p* < 0.001, ** 0.001 < *p* < 0.01, * 0.01 < *p* < 0.05, . 0.05 < *p* < 0.1.

| Assay name | Freshwater | Seawater | *t*-value | *p*-value | Signif. |
| --- | --- | --- | --- | --- | --- |
| ACTB_v1 | 0.781499 | 0.614146 | 0.454184 | 0.656659 |  |
| CA4_v1 | -0.16458 | 1.665436 | -5.67612 | 7.47E-05 | *** |
| CCL19_v1 | -1.1124 | -2.10943 | 2.783734 | 0.014903 | * |
| CCL4_v1 | 0.324899 | -2.61555 | 7.859551 | 1.13E-05 | *** |
| CFTR.I_v1 | 0.367682 | 1.632086 | -1.77163 | 0.107445 |  |
| CLEC4M_v1 | -0.72391 | -0.34098 | -0.60563 | 0.5545 |  |
| CYP2K1_v2 | 0.229148 | 0.581438 | -0.42066 | 0.680431 |  |
| EEF2_v1 | 1.00145 | 1.11542 | -0.5718 | 0.576693 |  |
| EXO1_v1 | 0.365374 | 0.453229 | -0.49978 | 0.625311 |  |
| FKBP5_v1 | -0.03752 | 0.537554 | -1.37563 | 0.196721 |  |
| FMNL1_v1 | 0.401456 | 0.076089 | 0.927794 | 0.369291 |  |
| GHR1_v1 | 0.5083 | 0.260142 | 0.467005 | 0.647695 |  |
| HBA_v1 | 1.379431 | 1.06952 | 0.756477 | 0.463339 |  |
| HBAt_v1 | -0.64149 | -1.82298 | 1.600008 | 0.132927 |  |
| IFI44_v1 | 0.128963 | -0.9276 | 3.608317 | 0.002892 | ** |
| IL12B_v1 | 1.002072 | 0.131237 | 1.740927 | 0.103722 |  |
| MCM4_v1 | 0.276983 | 0.247993 | 0.131698 | 0.897428 |  |
| MPC1_v1 | 0.889465 | 0.567753 | 0.939307 | 0.363611 |  |
| MS4A4A_v1 | 0.973487 | 0.1996 | 2.029614 | 0.061889 | . |
| NAMPT_v1 | 0.92058 | -0.05864 | 2.503539 | 0.025465 | * |
| NDUFB2_v1 | 1.034136 | 0.233148 | 2.567294 | 0.022735 | * |
| NDUFB4_v1 | 0.708509 | 0.322051 | 1.252024 | 0.231117 |  |
| NKAa1.a_v2 | 1.05694 | -1.17179 | 7.392296 | 1.28E-05 | *** |
| NKAa1.b_v2 | 0.312624 | 0.763602 | -1.44866 | 0.169462 |  |
| NR3C1_v1 | 0.876391 | 0.443096 | 1.640321 | 0.123257 |  |
| PLK2_v2 | 1.101163 | 0.811895 | 0.877102 | 0.395861 |  |
| PRLR_v1 | 1.421913 | -0.68146 | 6.365303 | 3.24E-05 | *** |
| RGS21_v1 | -0.32839 | 1.33284 | -6.60176 | 1.19E-05 | *** |
| RHAG_v1 | 1.739602 | 0.695542 | 3.181861 | 0.006896 | ** |
| RPL31_v1 | 0.646804 | 0.496804 | 0.554798 | 0.589087 |  |
| SLC16A10_v1 | 1.488335 | 1.127572 | 0.762769 | 0.460651 |  |
| THRB1_v2 | 1.371714 | 0.648913 | 1.865934 | 0.083299 | . |
| TRA_v1 | 0.125936 | 0.032976 | 0.305942 | 0.764628 |  |
| TSPO_v2 | 1.123814 | 0.046151 | 2.58888 | 0.021507 | * |
| TUBA8L2_v1 | 0.132761 | -0.18029 | 1.625875 | 0.126282 |  |
| UBA1_v1 | 0.745251 | -0.2679 | 3.094738 | 0.008873 | ** |
| WAS_v1 | 0.272223 | -0.28009 | 2.243392 | 0.041572 | * |

Table S12. Summary of body variable correlations with smoltification gene expression patterns for the four groups.

Presented are the Pearson correlations (*r*) with *p*-values in brackets for body variables and smoltification gene expression patterns (PC1 and PC2) using Coho salmon, Sockeye salmon, stream-type Chinook salmon, and ocean-type Chinook salmon. Smoltification gene expression patterns (PC1 and PC2), using the top 10 biomarkers, for each group are displayed in Figure 4 in the main text.

| Body variable | Coho salmon | | Sockeye salmon | | Chinook salmon (s-t) | | Chinook salmon (o-t) | |
| --- | --- | --- | --- | --- | --- | --- | --- | --- |
|  | PC1 | PC2 | PC1 | PC2 | PC1 | PC2 | PC1 | PC2 |
| Length | 0.47 (<0.001) | 0.27 (0.001) | 0.72 (<0.001) | -0.32 (0.004) | 0.25 (0.005) | -0.06 (0.475) | 0.77 (<0.001) | -0.44 (<0.001) |
| Mass | 0.44 (<0.001) | 0.19 (0.016) | 0.69 (<0.001) | -0.24 (0.032) | 0.22 (0.011) | -0.13 (0.131) | 0.74 (<0.001) | -0.38 (<0.001) |
| Condition | -0.13 (0.119) | -0.27 (0.001) | -0.16 (0.157) | 0.34 (0.002) | -0.05 (0.549) | -0.24 (0.005) | 0.52 (<0.001) | -0.14 (0.061) |
|  |  |  |  |  |  |  |  |  |
| Skin pigmentation |  |  |  |  |  |  |  |  |
| Posterior brightness | 0.18 (0.083) | 0.03 (0.749) |  |  |  |  | -0.17 (0.082) | 0.29 (0.003) |
| Anterior brightness | 0.07 (0.480) | 0.38 (<0.001) |  |  |  |  | 0.17 (0.094) | 0.29 (0.003) |
| Body yellowness | -0.19 (0.075) | 0.01 (0.941) |  |  |  |  | -0.03 (0.798) | -0.31 (0.002) |
| Caudal fin darkness | 0.33 (0.001) | 0.22 (0.033) |  |  |  |  | 0.26 (0.010) | 0.16 (0.107) |
|  |  |  |  |  |  |  |  |  |
| Morphology |  |  |  |  |  |  |  |  |
| Streamlined to truncated shape | -0.25 (0.017) | -0.38 (<0.001) |  |  |  |  | 0.03 (0.766) | 0.49 (<0.001) |
| Caudal peduncle length | -0.26 (0.011) | 0.17 (0.108) |  |  |  |  | 0.48 (<0.001) | 0.10 (0.326) |
